# Association between plausible genetic factors and weight loss from GLP1-RA and bariatric surgery

**DOI:** 10.1038/s41591-025-03645-3

**Published:** 2025-04-18

**Authors:** Jakob German, Mattia Cordioli, Veronica Tozzo, Sarah Urbut, Kadri Arumäe, Roelof A. J. Smit, Jiwoo Lee, Josephine H. Li, Adrian Janucik, Yi Ding, Akintunde Akinkuolie, Henrike O. Heyne, Andrea Eoli, Chadi Saad, Yasser Al-Sarraj, Rania Abdel-latif, Shaban Mohammed, Moza Al Hail, Alexandra Barry, Zhe Wang, Tatiana Cajuso, Andrea Corbetta, Pradeep Natarajan, Samuli Ripatti, Anthony Philippakis, Lukasz Szczerbinski, Bogdan Pasaniuc, Zoltán Kutalik, Hamdi Mbarek, Ruth J. F. Loos, Uku Vainik, Andrea Ganna

**Affiliations:** 1 Institute for Molecular Medicine Finland (FIMM), University of Helsinki, Helsinki, Finland; 2 Eric and Wendy Schmidt Center, Broad Institute of MIT and Harvard, Cambridge, MA USA; 3 Broad Institute of MIT and Harvard, Cambridge, MA USA; 4 Department of Computational Medicine, David Geffen School of Medicine at UCLA, Los Angeles, CA USA; 5 Division of Cardiovascular Medicine, Massachusetts General Hospital, Boston, MA USA; 6 Center for Genomic Medicine, Massachusetts General Hospital, Boston, MA USA; 7 Cardiovascular Research Center, Massachusetts General Hospital, Boston, MA USA; 8 Institute of Psychology, University of Tartu, Tartu, Estonia; 9 The Charles Bronfman Institute for Personalized Medicine, Icahn School of Medicine at Mount Sinai, New York, NY USA; 10 Department of Environmental Medicine, Icahn School of Medicine at Mount Sinai, New York, NY USA; 11 Novo Nordisk Foundation Center for Basic Metabolic Research, University of Copenhagen, Copenhagen, Denmark; 12 Novo Nordisk Foundation Center for Genomic Mechanisms of Disease, Broad Institute of MIT and Harvard, Cambridge, MA USA; 13 Diabetes Unit, Massachusetts General Hospital, Boston, MA USA; 14 Program in Medical & Population Genetics, Broad Institute of Harvard and MIT, Cambridge, MA USA; 15 Harvard Medical School, Boston, MA USA; 16 Center for Digital Medicine, Medical University of Bialystok, Bialystok, Poland; 17 Hasso Plattner Institute, University of Potsdam, Potsdam, Germany; 18 Hasso Plattner Institute for Digital Health at Mount Sinai, Icahn School of Medicine at Mount Sinai, New York, NY USA; 19 Qatar Genome Program, Qatar Precision Health Institute, Qatar Foundation, Doha, Qatar; 20 Department of Pharmacy, Hamad Medical Corporation, Doha, Qatar; 21 Harvard T.H. Chan School of Public Health, Boston, MA USA; 22 Department of Epidemiology, University of Alabama at Birmingham, Birmingham, AL USA; 23 Department of Pathology, Dana-Farber Cancer Institute and Harvard Medical School, Boston, MA USA; 24 Health Data Science Centre, Human Technopole, Milan, Italy; 25 MOX - Laboratory for Modeling and Scientific Computing, Department of Mathematics, Politecnico di Milano, Milan, Italy; 26 Personalized Medicine, Mass General Brigham, Boston, MA USA; 27 Department of Medicine, Harvard Medical School, Boston, MA USA; 28 Department of Public Health, Clinicum, University of Helsinki, Helsinki, Finland; 29 Analytic & Translational Genetics Unit, Massachusetts General Hospital, Harvard Medical School, Boston, MA USA; 30 Programs in Metabolism and Medical & Population Genetics, Broad Institute of MIT and Harvard, Cambridge, MA USA; 31 Department of Endocrinology, Diabetology and Internal Medicine, Medical University of Bialystok, Bialystok, Poland; 32 Clinical Research Centre, Medical University of Bialystok, Bialystok, Poland; 33 Department of Genetics, Perelman School of Medicine, University of Pennsylvania, Pennsylvania, USA; 34 Department of Pathology and Laboratory Medicine, David Geffen School of Medicine at UCLA, Los Angeles, CA USA; 35 Department of Human Genetics, David Geffen School of Medicine at UCLA, Los Angeles, CA USA; 36 Institute of Biomedical Informatics, Perelman School of Medicine, University of Pennsylvania, Philadelphia, PA USA; 37 University Center for Primary Care and Public Health, Lausanne, Switzerland; 38 Department of Computational Biology, University of Lausanne, Lausanne, Switzerland; 39 Swiss Institute of Bioinformatics, Lausanne, Switzerland; 40 Estonian Genome Centre, Institute of Genomics, University of Tartu, Tartu, Estonia; 41 Department of Neurology and Neurosurgery, McGill University, Montreal, Quebec Canada

**Keywords:** Genetics research, Obesity

## Abstract

Obesity is a major public health challenge. Glucagon-like peptide-1 receptor agonists (GLP1-RA) and bariatric surgery (BS) are effective weight loss interventions; however, the genetic factors influencing treatment response remain largely unexplored. Moreover, most previous studies have focused on race and ethnicity rather than genetic ancestry. Here we analyzed 10,960 individuals from 9 multiancestry biobank studies across 6 countries to assess the impact of known genetic factors on weight loss. Between 6 and 12 months, GLP1-RA users had an average weight change of −3.93% or −6.00%, depending on the outcome definition, with modest ancestry-based differences. BS patients experienced −21.17% weight change between 6 and 48 months. We found no significant associations between GLP1-RA-induced weight loss and polygenic scores for body mass index or type 2 diabetes, nor with missense variants in *GLP1R*. A higher body mass index polygenic score was modestly linked to lower weight loss after BS (+0.7% per s.d., *P* = 1.24 × 10^−4^), but the effect attenuated in sensitivity analyses. Our findings suggest known genetic factors have limited impact on GLP1-RA effectiveness with respect to weight change and confirm treatment efficacy across ancestry groups.

## Abstract (web summary)

The authors found, after analyzing 10,960 individuals from 9 multiancestry biobanks across 6 countries, that genetic factors previously associated with BMI have limited impact on GLP-1 receptor agonist-induced weight loss.

## Main

The obesity epidemic presents a substantial public health burden, with 75% of adults with obesity attempting weight loss but few achieving lasting success^1–3^. Amid these challenges, GLP1-RA have emerged as a promising solution. Discovered in the 1980s, glucagon-like peptide-1 (GLP1) is linked to the incretin effect, promoting insulin release from pancreatic β cells, thus regulating blood sugar levels^4^. This led to approval of the first GLP1-RA for treatment of type 2 diabetes (T2D) in 2005, followed by approval for obesity treatment in 2014 after its association with reduced food intake was established^5^.

Clinical trials demonstrated weight loss effects of 6–16% with liraglutide and semaglutide, driving a global rise in GLP1-RA use^6–8^. Additional trials have revealed cardiovascular^9,10^ and renal benefits^11^, with ongoing trials underway^12^. By 2030, an estimated 30 million people in the United States (~9% of the population) are expected to use GLP1-RA drugs, highlighting their role in addressing obesity and expanding therapeutic applications^13,14^.

Meanwhile, BS remains a durable and effective treatment for individuals with severe obesity and metabolic comorbidities (body mass index (BMI) ≥ 35 kg m^−^^2^)^15,16^. Meta-analyses of randomized controlled trials and observational studies report an average weight loss of 26 kg, with one trial showing 25% total weight loss after 5 years for sleeve gastrectomy (SG) and Roux-en-Y gastric bypass (RYGB)^17,18^. Although BS carries risks such as nutrient malabsorption, large-scale observational studies have demonstrated remarkable long-term benefits, including reduced cardiovascular outcomes, lower all-cause mortality and higher remission rates of T2D, hypertension and hyperlipidemia^19–22^.

Despite the robust weight loss effects observed with both GLP1-RA treatment and BS, there exists heterogeneity in their effectiveness. Up to 20% of BS patients may not achieve the desired weight loss in the first year or may regain weight in two years^23,24^. Variability in GLP1-RA’s glycemic effects has been linked to clinical factors like low β cell function, C-peptide levels and genetic variants^25–29^. However, aside from T2D, sex and baseline weight, factors driving differences in weight loss responses remain poorly understood^30–32^. In addition, differences in patient adherence may further contribute to this variability^33^.

Genetics has been considered a potential factor for heterogeneity in weight loss treatments, but its role remains largely unexplored^30^. A previous study of 57 women found a weak association between a common *GLP1R* missense variant and GLP1-RA response^27,34^. Although more well-powered studies have been conducted in BS patients^35,36^, no consistent genome-wide associations for weight loss have been identified. However, a polygenic score (PGS) for BMI was significantly linked to weight loss after biliopancreatic diversion with duodenal switch in 865 patients^37^.

Finally, there has been limited exploration into the effectiveness of these obesity treatments across different genetic ancestries and across various healthcare settings^6,38^.

In our study we seek to: (1) characterize the observed GLP1-RA and BS body weight-lowering effects using real-world data from 10,960 individuals across nine biobanks in six countries, with six major continental ancestry groups; (2) identify whether plausible genetic factors (PGS for BMI and T2D, and coding variants in *GLP1R*, *PCSK1* and *APOE* genes) associate with heterogeneity in GLP1-RA and BS weight loss effects, and thus identify patient groups that will benefit most from such treatments; (3) compare the effects of GLP1-RA treatment and BS on weight loss across major continental ancestry groups.

## Results

### GLP1-RA and BS weight loss across ancestries

We investigated changes in body weight associated with GLP1-RA treatment and BS across nine biobank studies: Helsinki University Hospital (HUS, Finland; *n* = 633), Estonian Biobank (ESTBB; *n* = 464), UK Biobank (UKBB, United Kingdom; *n* = 810), All of Us (AoU, United States; *n* = 559), BioMe Biobank (BioMe, United States; *n* = 2,170), Mass General Brigham Biobank (MGBB, United States; *n* = 2,141), Atlas Biobank (UCLA-ATLAS, United States; *n* = 1,445), Qatar Biobank (QBB, Qatar; *n* = 2,383) and Bialystok Bariatric Surgery Study (BBSS, Poland, *n* = 355). We included individuals that were at least 18 years of age when starting a GLP1-RA treatment or undergoing BS. Individuals undergo GLP1-RA treatment for different medical reasons, not only for weight loss. We defined GLP1-RA usage based on prescription or purchase data (ATC codes starting with A10B*; including exenatide, liraglutide, lixisenatide, albiglutide, dulaglutide, semaglutide and beinaglutide) covering at least 12 months, and excluding individuals with indication of treatment discontinuation within this time window. For BS, we considered individuals undergoing the following procedures: RYGB, SG, adjustable gastric band, vertical banded gastroplasty or biliopancreatic diversion with duodenal switch. More information can be found in the Methods.

**Extended Data Fig. 1:**
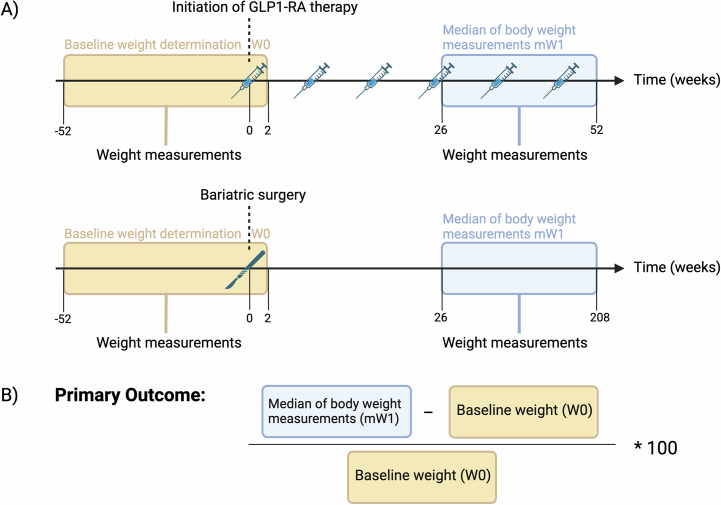
Study Design. **a**, Eligibility criteria are displayed for the GLP1-RA analysis and the BS analysis. The axis represents time in weeks. The highlighted areas depict the time intervals for determining the baseline weight (W0) and final weight (mW1), defined as the median of body weight measurements in the given time interval. Detailed information on the eligibility criteria can be found in the Methods. **b**, Outcome definition for our primary model. Detailed information can be found in the Methods. Created in BioRender. German, J. (2025) https://BioRender.com/i42b370.

We included only individuals with an initial body weight measurement recorded in the 12 months before baseline (treatment initiation or surgery) and a follow-up measurement taken 6–12 months after baseline for GLP1-RA or within 48 months for BS. In case of multiple measurements in this time frame, we considered their median (Extended Data Fig. 1). We defined our main outcome of interest as the percentage change in body weight, calculated as the difference between the second body weight measurement (or median of multiple measurements) and the initial one, divided by the initial measurement.

Our study population included a total of 6,750 GLP1-RA users and 4,210 individuals undergoing BS (baseline characteristics are reported in Table 1). Overall, GLP1-RA users: (1) had a lower proportion of women (61.0% weighted average across studies) when compared with BS patients (73.1% weighted average across studies); (2) were on average older (57.30 versus 46.50 years); and (3) had a lower initial body weight (99.80 kg versus 116.96 kg). The proportion of individuals diagnosed with T2D at time of GLP1-RA initiation was ≥48% across all studies and major continental ancestry groups.

**Table 1:** Baseline characteristics for 6,750 GLP1-RA users and 4,210 patients undergoing BS included in our study population

Study	Ancestry	No. of individuals	Proportion of women (%)	Mean body weight at baseline (s.d.)	Mean age at baseline (s.d.)	T2D prevalence (%)	Most common GLP1-RA type	Proportion of semaglutide (%)
GLP1-RA								
HUS	EUR	255	68.00	113.70 (22.90)	53.79 (13.52)	65.49	Semaglutide	60.40
ESTBB	EUR	191	62.30	109.18 (20.3)	56.95 (10.96)	89.01	Semaglutide	72.77
UKBB	EUR	614	44.30	105.00 (19.90)	60.50 (7.15)	99.80	Liraglutide	0.00
AoU	EUR	117	64.96	106.66 (24.18)	57.01 (11.03)	82.91	Liraglutide	11.97
	AFR	91	83.52	104.8 (25.01)	54.57 (11.93)	92.31	Liraglutide	18.68
MGBB	EUR	857	57.01	99.99 (21.48)	59.88 (12.17)	56.33	Semaglutide	40.72
	AFR	156	54.32	104.65 (23.80)	54.31 (11.91)	60.24	Dulaglutide	36.14
BioMe	AMR	614	66.45	91.19 (22.45)	59.07 (11.29)	67.75	Dulaglutide	29.32
	AFR	728	69.78	101.49 (25.42)	57.00 (12.24)	69.50	Dulaglutide	29.12
	EUR	369	44.44	98.26 (21.93)	60.32 (11.57)	47.97	Semaglutide	37.13
	SAS	77	50.65	83.79 (20.10)	56.48 (12.59)	70.13	Semaglutide	41.56
UCLA-ATLAS	AFR	133	64.00	101.6 (23.20)	61.00 (13.00)	91.00	Semaglutide	57.10
	AMR	336	66.00	94.40 (22.60)	56.00 (13.00)	84.00	Liraglutide	26.50
	EAS	120	47.00	81.50 (16.30)	60.00 (13.00)	92.00	Semaglutide	61.70
	EUR	856	53.00	101.60 (22.00)	63.00 (13.00)	80.00	Semaglutide	67.80
QBB	MID	1,236	72.17	97.68 (24.54)	49.52 (10.75)	75.97	Liraglutide	3.64
Bariatric surgery								
HUS	EUR	378	81.00	113.63 (19.5)	49.90 (9.39)	37.04		
ESTBB	EUR	273	76.56	121.24 (20.57)	44.72 (10.07)	28.21		
UKBB	EUR	196	69.40	121.00 (29.70)	55.50 (8.21)	50.00		
AoU	EUR	168	81.55	121.32 (29.88)	50.74 (12.62)	18.45		
	AFR	183	89.62	127.23 (32.55)	46.09 (11.67)	48.63		
MGBB	EUR	978	71.06	121.21 (26.52)	49.37 (12.16)	39.98		
	AFR	150	86.67	122.89 (23.92)	43.68 (11.67)	42.67		
BioMe	AMR	143	79.72	112.36 (20.81)	44.62 (11.48)	30.07		
	AFR	188	82.98	124.88 (25.63)	45.35 (12.16)	29.26		
	EUR	51	70.59	120.23 (25.29)	49.51 (13.34)	17.65		
QBB	MID	1,147	69.49	102.14 (23.18)	41.66 (10.66)	40.02		
BBSS	EUR	355	56.06	138.48 (27.07)	47.10 (7.11)	35.21		

For each cohort and major continental ancestry group we report the total number of individuals eligible for analysis, the proportion of females, the mean (s.d.) body weight (kg) at baseline, the mean (s.d.) age (years) at baseline, the prevalence of T2D and, for GLP1-RA users only, the most commonly used type of medication and the proportion of semaglutide users.

**Fig. 1:**
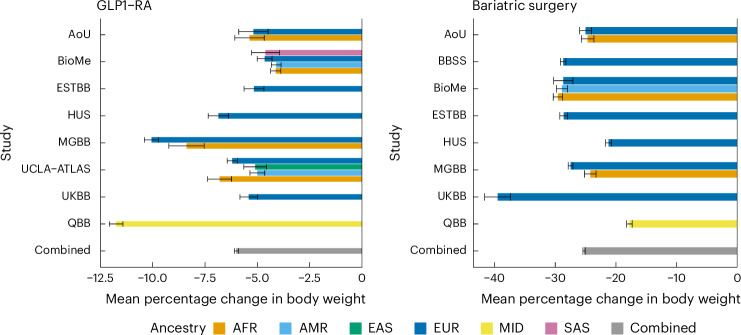
Average percentage change in body weight for 6,750 GLP1-RA users and 4,210 BS patients. Bars represent the mean percentage change in body weight in each study and ancestry, and the overall combined weighted mean change. Error bars represent standard errors. Total *n* for GLP1-RA = 6,750, total *n* for BA = 4,210, sample size per each cohort or ancestry can be found in Table 1. Source data

Among GLP1-RA users, we observed an average body weight change (Fig. 1) across studies of −3.93% (ranging from −1.08% to −7.07%), in line with results from observational studies^39,40^. Among BS patients, we observed an average change of −21.17% (−15.00% to −27.72%).

### Demographic factors linked to GLP1-RA and BS weight change

**Extended Data Fig. 2:**
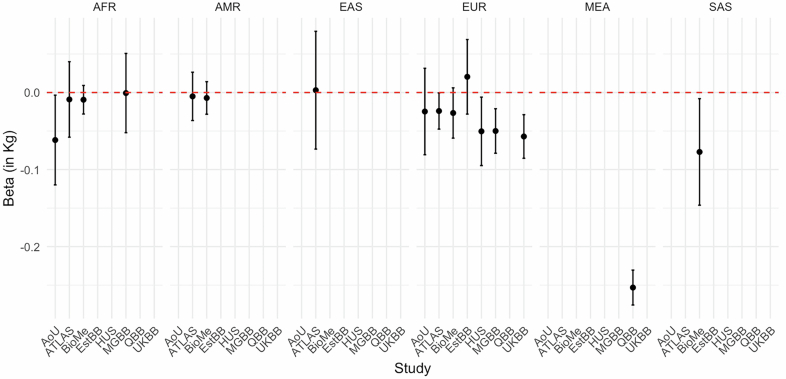
Effect sizes of baseline weight on weight loss amongst GLP1-RA users across studies by ancestries. Points represent beta coefficients in Kg and error bars represent the 95% confidence intervals. Negative beta coefficients indicate that higher baseline weight is associated with greater weight loss, while positive beta coefficients indicate the opposite. Total N = 6 750, sample size per each cohort/ancestry can be found in Table 1. AoU: All of Us, ATLAS: UCLA-Atlas Biobank, BioMe: BioMe Biobank, ESTBB: Estonian Biobank, HUS: Helsinki University Hospital, MGBB: Mass General Brigham Biobank, QBB: Qatar Biobank, UKBB: UK Biobank, AFR: African, AMR: admixed American, EAS: East Asian, EUR: European, MID: Middle Eastern, SAS: South Asian. Source data

**Extended Data Fig. 3:**
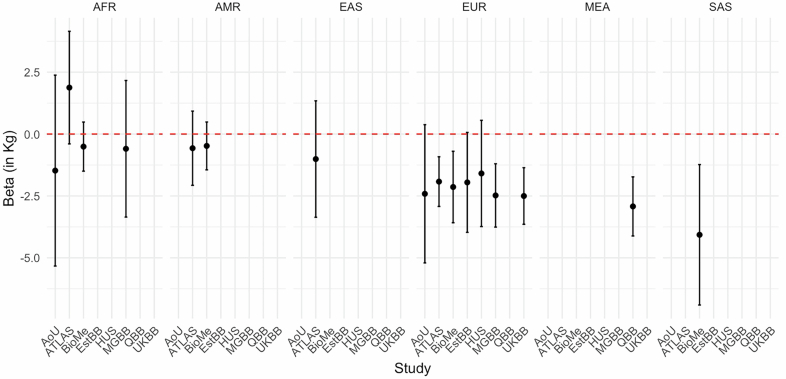
Effect sizes of sex on weight loss amongst GLP1-RA users across studies by ancestries. Points represent beta coefficients in Kg and error bars represent the 95% confidence intervals. Negative beta coefficients indicate that female sex is associated with greater weight loss, while positive beta coefficients indicate the opposite. Total N = 6 750, sample size per each cohort/ancestry can be found in Table 1. AoU: All of Us, ATLAS: UCLA-Atlas Biobank, BioMe: BioMe Biobank, ESTBB: Estonian Biobank, HUS: Helsinki University Hospital, MGBB: Mass General Brigham Biobank, QBB: Qatar Biobank, UKBB: UK Biobank, AFR: African, AMR: admixed American, EAS: East Asian, EUR: European, MID: Middle Eastern, SAS: South Asian. Source data

**Extended Data Fig. 4:**
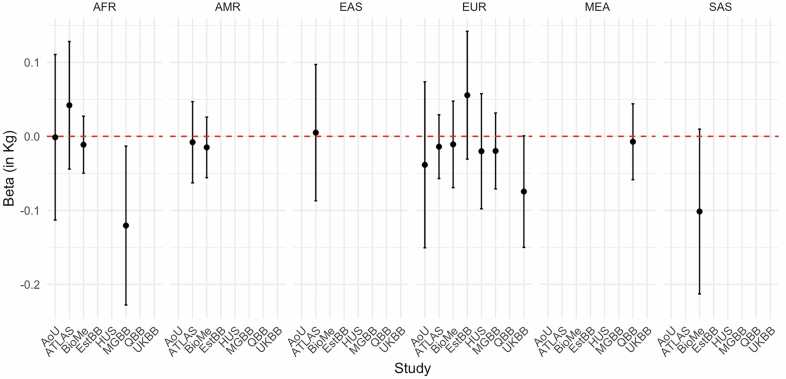
Effect sizes of age at treatment initiation on weight loss amongst GLP1-RA users across studies by ancestries. Points represent beta coefficients in Kg and error bars represent the 95% confidence intervals. Negative beta coefficients indicate that baseline age is associated with greater weight loss, while positive beta coefficients indicate the opposite. Total N = 6 750, sample size per each cohort/ancestry can be found in Table 1. AoU: All of Us, ATLAS: UCLA-Atlas Biobank, BioMe: BioMe Biobank, ESTBB: Estonian Biobank, HUS: Helsinki University Hospital, MGBB: Mass General Brigham Biobank, QBB: Qatar Biobank, UKBB: UK Biobank, AFR: African, AMR: admixed American, EAS: East Asian, EUR: European, MID: Middle Eastern, SAS: South Asian. Source data

We explored the effect of baseline characteristics on body weight changes associated with GLP1-RA treatment and BS. We fitted a multivariable linear model with percentage change in body weight as outcome and baseline body weight, sex, age at baseline, the first 20 principal genetic components and medication type (for GLP1-RA users only) as predictors. Each cohort was analyzed separately, stratifying by major continental ancestry group, and the effects were then meta-analyzed (Table 2). Baseline body weight was significantly associated with higher weight loss for both GLP1-RA and BS treatments (*β*_GLP1-RA_ = −0.05% weight change compared with baseline, *P* = 1.63 × 10^−35^; *β*_BS_ = −0.14% weight change compared with baseline, *P* = 5.49 × 10^−69^) and women had a significantly higher weight loss for both treatments (*β*_GLP1-RA_ = −1.54% weight change compared with baseline, *P* = 2.47 × 10^−16^; *β*_BS_ = −2.66% weight change compared with baseline, *P* = 6.83 × 10^−10^). Being older at treatment initiation was only associated with less weight loss for BS (*β*_BS_ = 0.15% weight change compared with baseline, *P* = 2.54 × 10^−15^). The effects of baseline weight and sex on weight loss were highly heterogeneous across ancestries and studies (heterogeneity (*I*^2^) for GLP1-RA = 0.99 and 0.83 for baseline weight and sex, respectively) (Extended Data Figs. 2–4 and Supplementary Tables 1 and 2).

**Table 2:** Effect of baseline weight, sex and age at baseline of percentage weight changes associated with GLP1-RA and BS

Variable	GLP1-RA			Bariatric surgery		
	Effect on percentage weight change	Standard error	*P* value	Effect on percentage weight change	Standard error	*P* value
Baseline weight (kg)	−0.05	0.004	1.63 × 10^−35^	−0.14	0.008	5.49 × 10^−69^
Sex (female)	−1.54	0.188	2.74 × 10^−16^	−2.66	0.431	6.83 × 10^−10^
Age at baseline (years)	−0.02	0.008	0.052	0.15	0.016	9.85 × 10^−19^

Coefficients are derived from a multivariable linear model including the three variables described in the table and the first 20 principal genetic components and medication type (for GLP1-RA users only). Negative effects indicate larger weight loss. *P* values are two-sided and were calculated by dividing the coefficient values by their standard errors and observing the probability mass corresponding to equal or more extreme values from both tails of Student’s *t* distribution.

### Ancestry has marginal impact on GLP1-RA and BS weight loss

We evaluated whether the effect of GLP1-RA- and BS-associated weight loss was significantly different across genetically defined continental ancestry groups after accounting for differences in weight at baseline, age, sex and, for GLP1-RA only, medication type (Table 3). Only multiancestry biobanks (AoU, BioMe, MGBB, UCLA-ATLAS) were included in this analysis (*n* = 6,315). Compared with individuals of European ancestry, all other ancestry groups had a lower change in body weight. However, this effect was statistically significant only among GLP1-RA users of African (*β* = 0.76% weight change from baseline weight, *P* = 0.02) and admixed American (*β* = 0.87% weight change from baseline weight, *P* = 3 × 10^−3^) ancestries.

**Table 3:** Associations between weight change and major continental ancestry groups

Major continental ancestry group	Effect on percentage weight change	Standard error	*P* value
GLP1-RA			
AFR	0.758	0.327	0.021
AMR	0.874	0.295	0.003
EAS	1.093	0.664	0.100
SAS	0.118	0.759	0.876
BS			
AFR	1.688	1.308	0.197
AMR	−0.542	1.428	0.704

Meta-analysis effect sizes, standard errors and *P* values for the association between weight change and major continental ancestry groups. Coefficients are derived from a multivariable model including percentage weight change as the outcome and ancestry as a categorical variable (with EUR as the reference category), and adjusting for sex, age and weight at baseline and, for GLP1-RA only, medication type. Negative effects indicate higher weight loss. *P* values are two-sided and were calculated by dividing the coefficient values by their standard errors and observing the probability mass corresponding to equal or more extreme values from both tails of Student’s *t* distribution.

### Plausible genetic factors show no link to GLP1-RA weight loss

We investigated the effect of genetic exposures with plausible effects on body weight changes. Genetic exposures were selected upon discussion between authors and before initiating analyses. First, we considered PGS for BMI based on previous observation that a PGS was associated with BMI trajectories^41,42^. We also consider the only replicated genome-wide variant associated with BMI change in the largest study of BMI trajectories (rs429358 in *APOE*)^41^. Second, we considered a PGS for T2D based on the observation that biomarkers of T2D were associated with effectiveness of GLP1-RA^26,43^. We confirmed that both PGS for BMI and T2D were significantly associated with their respective traits across all major continental ancestry groups (Supplementary Table 3) although the effects were larger in individuals of European ancestry.

Third, we considered 13 single nucleotide polymorphisms (SNPs) in the *GLP1R* and *PCSK1* genes. We considered all missense variants in the *GLP1R* gene with at least 1% minor allele frequency (MAF) in at least one of major continental ancestry group, as per gnomAD (v.4.1)^44^, three of which have been functionally characterized as gain or loss of function (Supplementary Table 4)^45^. We also considered two nonsynonymous variants in the *PCSK1* gene that have been associated with BMI variation in the population^46^. Finally, we sought to replicate the effect of two variants associated with weight loss in one of the largest studies of BS^36^ (Methods). After analyses, two missense variants in the *GLP1R* genes rs2295006 and rs201672448 were too rare to be tested across studies and were not considered further.

**Fig. 2:**
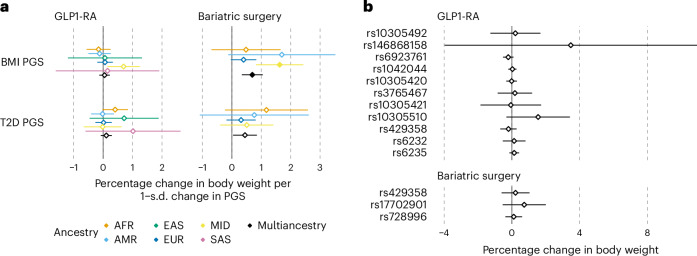
Effect of 15 genetic exposures on body weight changes associated with GLP1-RA treatment and BS. **a**, Ancestry-specific and multiancestry meta-analysis effect sizes for the association between percentage change in body weight and PGS for BMI and T2D. Dots represent the estimated percentage change in body weight per 1 s.d. change in PGS, error bars represent the 95% confidence interval (CI) for the estimate. **b**, Multiancestry meta-analysis effect sizes for the association between percentage change in body weight and genotype at each locus. Dots represent the estimated percentage change in body weight, error bars represent the 95% CI for the estimate. For **a** and **b**, coefficients are estimated from a linear regression model, separately for each genetic exposure. Full dots represent statistical significance at *P* < 0.004 (Bonferroni adjusted for 14 independent tests). *P* values are two-sided and were calculated by dividing the coefficient values by their standard errors and observing the probability mass corresponding to equal or more extreme values from both tails of Student’s *t* distribution. Exact *P* values can be found in Supplementary Tables 5 and 6. Total sample size is 6,750 for GLP1-RA and 4,210 for BS. Sample size per ancestry can be found in Table 1. Source data

We estimated the effect of each genetic exposure on weight change in each cohort and major continental ancestry groups separately, fitting a linear model adjusted for baseline body weight, sex, age at baseline, first 20 principal genetic components and medication type (for GLP1-RA users only), and we subsequently combined the effects through ancestry-specific and multiancestry meta-analyses (Fig. 2 and Supplementary Tables 5 and 6). We did not adjust for T2D diagnosis because we considered it a potential mediator between the genetic exposure and weight change outcome.

Among GLP1-RA users, we did not observe any significant genetic exposure associated with weight loss after multiple-testing correction (*P* < 0.004, Bonferroni correction for 13 exposures tested).

We further tested whether the lack of associations observed was possibly due to high effect-size heterogeneity across ancestries, or to a lack of statistical power. We did not observe any significant heterogeneity across ancestries for any of the genetic exposure (average *I*^2^ across genetic exposures for GLP1-RA was 0.06) (Supplementary Table 7). Moreover, given the current sample size for GLP1-RA users (*n* = 6,750) our power calculation showed we would have at least 80% power to detect a statistically significant effect (at *P* < 0.05) of at least a 0.3% change in body weight for 1 s.d. change in the PGS, or for SNPs with an MAF of at least 1% (Methods).

### A higher BMI PGS associates with lower weight loss due to BS

Among BS patients, a higher PGS for BMI was significantly associated with lower weight loss (*β*_BMI PGS_ = 0.70% weight change compared with baseline for 1 s.d. change in the PGS, *P* = 1.24 × 10^−4^) after multiple-testing correction (*P* < 0.01, Bonferroni correction for five exposures tested), in the opposite direction than observed for weight at baseline (Fig. 2 and Table 2). A higher PGS for T2D was nominally associated with lower weight loss (*β*_T2D PGS_ = 0.45% weight change compared with baseline for 1 s.d. change in the PGS, *P* = 0.03).

We observed significant heterogeneity in the association between BMI PGS and weight loss across studies (*I*^2^ = 0.64) (Supplementary Table 8).

### Sensitivity analyses

To assess the robustness of our findings, we conducted a series of sensitivity analyses (Supplementary Methods and Supplementary Results).

First, we consider different outcome definitions. We shortened the follow-up period for BS to 12 months to make it comparable with that of GLP1-RA. We considered only participants with weight measurements available at least 30 days before BS to account for the possibility of substantial weight loss due to preoperative low-calorie diets. We used minimum rather than median weight during the follow-up period to assess the impact of a different definition of weight loss. This latter definition resulted in significant higher weight loss for GLP1-RA (−6.00% compared with −3.93% when using the median) (Supplementary Tables 9 and 10 and Supplementary Figs. 1 and 2). All analyses confirmed the associations observed in the primary analysis (Supplementary Tables 11–21 and Supplementary Figs. 1–12), although the effect of BMI PGS on weight loss due to BS was slightly reduced when considering a shortened follow-up period (0.43% per 1 s.d. change in the PGS, *P* = 0.03 compared with 0.70% in the primary model) (Supplementary Table 11 and Supplementary Figs. 3 and 4).

Second, we considered different covariate adjustment and performed subgroup analyses. To evaluate whether a T2D diagnosis independently affects weight loss, we adjusted the analyses for T2D status and observed similar results to the primary analyses (for BMI PGS in BS, the only statistically significant result, 0.59% per 1 s.d. change, *P* = 6.6 × 10^−4^ compared with 0.70% in the primary model) (Supplementary Tables 12 and 13 and Supplementary Fig. 5). We considered only semaglutide and liraglutide as GLP1-RA treatments to capture their specific use for weight loss. Although we observed a higher weight loss than considering all GLP1-RA treatments (−4.69% versus −3.93%) (Supplementary Fig. 6), we confirmed the lack of effect of BMI and T2D PGS on weight loss observed in primary analyses (Supplementary Table 14 and Supplementary Fig. 7). We stratified the BS analysis by the two major surgery procedures, RYGB and SG, allowing us to assess whether the observed effects varied by surgery type. Although both procedures showed similar weight loss effects (−22.30% and −22.10%, for RYGB and SG respectively) (Supplementary Figs. 10 and 11), we observed a significant effect of BMI PGS on weight change only for RYGB (0.83% weight change compared with baseline per 1 s.d. change in the PGS, P = 1.49 × 10^−4^ ((Supplementary Tables 16 and 17).

Third, we considered different statistical models. Previous works have argued that using percentage changes in weight loss pre versus post therapy is statistically inappropriate and inefficient^47–49^. Thus, we considered a model using post-treatment weight as outcome, including the same predictors as in the main model and an additional term modeling the interaction between baseline weight and each genetic exposure. We observed similar results as in the primary model (Supplementary Tables 18 and 19). A second potential issue with the primary model relates to adjusting longitudinal change phenotypes for the baseline trait^50,51^. In particular, when testing for the effect of genetic exposure on drug response outcomes, adjusting phenotype changes (for example, weight loss) for baseline levels (for example, weight at treatment initiation) can result in a false-positive association when genetic variants are also associated with baseline levels^52^. Removing baseline weight from the model led to a reduction in the effect of the BMI PGS on weight loss due to BS (0.15% per 1 s.d. change in the PGS, *P* = 0.420, compared with 0.70% in the primary model) (Supplementary Tables 20 and 21 and Supplementary Fig. 12).

## Discussion

In this study, we provide a comprehensive assessment of major demographic factors and plausible genetic factors on body weight changes associated with GLP1-RA use and BS. In line with previous work^30,53–56^, our main outcome of interest was the percentage change in body weight between 6 and 12 months after GLP1-RA treatment initiation as well as between 6 and 48 months after BS.

By considering studies from six different countries, we were able to highlight heterogeneity in the use of GLP1-RA across different healthcare systems. GLP1-RA users consisted mostly of individuals with T2D, reflecting their original use for treatment of this condition. Biobank studies covering Boston (MGBB), New York (BioMe), Los Angeles (UCLA-ATLAS) and Helsinki (HUS) had overall lower rates of T2D individuals compared with the other studies and lower weights at baseline, which, together with the availability of more updated electronic health records (EHR) data, suggest a larger number of individuals being prescribed GLP1-RA for weight loss. However, care should be taken in interpreting variation in the rate of T2D across biobanks because this might reflect different approaches used for capturing this diagnosis from the EHR as well as the different sampling strategies of biobanks.

The comparison between GLP1-RA and BS is informative because it highlights the younger age, higher weight and lower percentage of men undergoing BS compared with GLP1-RA users. The weight reduction was also approximately five times higher among BS patients compared with GLP1-RA users. The weight reduction among GLP1-RA users was also more heterogeneous across biobanks compared with BS, but in line with that reported previously in the literature using real-world data^28^ and lower than results from clinical trials^7,57^. The lower weight reduction observed among GLP1-RA users is partially influenced by use of the median postoperative or post-drug therapy weight, although we observed greater weight reduction when using minimum weight. However, our approach provides a more conservative and robust estimate, better accounting for the variability and potential uncertainty in clinical measurement data. Our findings also highlight a consistent effect of baseline body weight and sex on weight loss associated with both GLP1-RA treatment and BS, suggesting that individuals with higher weight at baseline, as well as women benefit more from both interventions. These findings underscore the importance of considering individual characteristics when designing weight loss interventions.

The main aim of this study was to evaluate the impact of genetic exposures on body weight changes induced by GLP1-RA or BS. At the current sample size, a genome-wide scan would have limited power to identify potential new signals. With 6,750 individuals, we would have at least 80% statistical power to identify, at a genome-wide significant threshold, a genetic variant with an allele frequency of ≥1% and an effect >0.3% weight change per s.d., being unlikely given previous effects of common variants on treatment response^52^. Instead, we tested hypotheses based on the largest genome-wide association studies of BMI and T2D and examined biologically plausible mutations in candidate genes. PGS represent the most powerful genetic predictors for BMI and T2D and we confirm their significant association with the corresponding trait across all major continental ancestry groups. Thus, lack of associations with these PGS would suggest that these interventions act independently of genetic predisposition to BMI or T2D, as explained by common genetic variants. Moreover, because the biological underpinning of weight change is partially distinct from weight at baseline, we also consider the few replicated genetic signals for BMI variability and BMI trajectories.

For BS, genetically higher BMI was associated with weight gain, opposite to the phenotypic association with initial body weight. This suggests that individuals with a stronger genetic predisposition to obesity may regain weight faster post surgery. Maintaining weight loss may require additional lifestyle modifications, behavioral therapy or adjunct pharmacologic treatments like GLP1-RA^58,59^.

By contrast, none of the genetic exposures were associated with weight loss among GLP1-RA users, after multiple-testing correction. These results were not attributable to high heterogeneity across studies or across major continental ancestry groups. Whether we directly tested the impact of genetic exposure on percentage body weight change or used post-treatment weight as outcome and tested the interaction between baseline weight and the genetic exposure, the results did not significantly change.

In addition, we conducted extensive sensitivity analyses to further examine the effect of BMI and T2D PGS on weight loss after BS and GLP1-RA treatment. These analyses included alternative definitions of weight outcomes, a shorter follow-up period and stricter inclusion criteria for BS, adjustments for T2D status, subgroup analysis for GLP1-RA medication and BS patients, and testing different statistical models. Across all these analyses, we did not observe a significant effect of BMI or T2D PGS on weight reduction following GLP1-RA treatment, in concordance with our primary analysis. Regarding the impact of BMI PGS on weight loss due to BS, we observed a reduction of effect in some of the sensitivity analyses, in particular when excluding baseline weight adjustment from the model. In this case, the directionality of the effect remained consistent; however, the association was no longer statistically significant. Further, no significant genetic effect was observed for SG. Notably, the QBB cohort contained only 23 individuals who underwent SG, resulting in a sample size too small to calculate standard errors for the estimates in the model. Consequently, the QBB cohort, and by extension, the Middle Eastern ancestry group, did not contribute to the SG subanalysis. The lack of separation between techniques and studies makes it difficult to discern whether the observed effects stem from surgical procedures, cohort characteristics or ancestry-related factors. Overall, our results showed that the considered genetic influence on weight loss in response to GLP1-RA treatment and BS may be too small to impact clinical decision-making or patient outcomes. Future studies should explore more powerful PGS or focus on individuals using GLP1-RA solely for weight loss.

Another aim was to compare GLP1-RA- and BS-associated weight loss across major continental ancestry groups. Ancestry can provide more useful information about population health than racial and ethnic categories because it is not directly influenced by geographic, cultural and sociopolitical forces^60^. Nonetheless, ancestry is also partially correlated with self-reported race and ethnicity and socioeconomic characteristics do differ across racial and ethnic groups. Overall, we found no large differences in GLP1-RA- and BS-associated weight loss between major continental ancestry groups. However, GLP1-RA users of African and admixed American ancestry showed significantly lower weight change than Europeans, after adjusting for sex, age, baseline weight and medication type. The effects were consistent in BioMe and UCLA-ATLAS for admixed American ancestry, and in AoU and MGBB for African ancestry. These differences may reflect socioeconomic factors, such as disparities in treatment access.

The main strength of our study is its size and diversity across countries, healthcare systems and ancestries. Yet, our study presents various limitations. First, we cannot be sure that all GLP1-RA users fulfilled their prescription, although this affects studies with prescription data more than those with purchase data. Nonetheless, weight loss was comparable across both study types. Moreover, to ensure weight changes reflected treatment effects, we included only measurements within one month of the last purchase or prescription. Second, we could not accurately estimate treatment adherence, but overcome this limitation by considering treatments of maximum 12 months’ duration, a period for which adherence has been observed to be around 65% (ref. ^53^). Third, we used ancestry labels based on major continental ancestry groups, while ancestry can be better characterized as a continuous quantity^60^. This discretization reflects pragmatic considerations to enable comparable analyses across the different studies. Fourth, the absence of a control group in the GLP1-RA analysis may limit distinguishing intentional from unintentional weight loss. However, large-scale real-world data and consistent sensitivity analysis support the robustness of our results. Fifth, although T2D medications like metformin can affect weight loss, our T2D-adjusted sensitivity analyses likely accounted for first-line treatments. Finally, we did not assess GLP1-RA dosing effects, because dose adjustments vary over time, higher doses do not consistently correlate with greater weight loss and inconsistent reporting across studies could introduce biases.

In conclusion, our study suggests that GLP1-RA treatments work equally well in individuals carrying common nonsynonymous mutations in the GLP1R gene and in individuals at high genetic risk for BMI and T2D. Although BS is slightly less effective in individuals with a higher BMI PGS, the effect (~0.7% lower weight loss per s.d. increase) may be not clinically substantial. Both interventions achieved sustained weight loss across ancestry groups, although socioeconomic factors linked to ancestry should be further studied to understand remaining differences.

## Methods

### Ethics statement

This study complies with all relevant ethical regulations and was conducted in accordance with the research permits and approvals granted by the respective institutions.

The INTERVENE Helsinki Biobank project was approved by the Helsinki University Central Hospital Ethics Board (HUS/230/2022 §69). The application meets the requirements for biobank research as defined by the Biobank Act (688/2012). The Bialystok Bariatric Surgery Study was approved by the Ethics Committee of the Medical University of Bialystok (approval number R-I-002/546/2015). This study was approved by the Institutional Review Board of the Icahn School of Medicine at Mount Sinai (23-00583). The Estonian Biobank (ESTBB) operates under the Human Genes Research Act, which has regulated its activities since 2000. Individual-level data analyses in ESTBB were conducted under ethical approvals 1.1-12/1409 and 1.1-12/2161 from the Estonian Committee on Bioethics and Human Research (Estonian Ministry of Social Affairs), using data according to release application 6-7/GI/18857 and 3-10/GI/11571 from the Estonian Biobank. The analysis in the Mass General Brigham Biobank (MGBB) was approved by the Mass General Brigham Institutional Review Board under protocol number 2018P001236. In Qatar Biobank all participants provided written informed consent for the biobank study and this study was approved by the Biobank Institutional Review Board number E-2024-QF-QBB-RES-QCC-00205-0280. UK Biobank data used in this study were obtained under approved application 78537.

For all other biobanks included in this study, additional ethical approval was not required as per their respective governance policies for secondary research using de-identified biobank data.

### Study population

In the current study, we included samples from 10,960 individuals from the following nine biobanks: HUS (Finland; *n* = 633), ESTBB (Estonia; *n* = 464), UKBB (United Kingdom; *n* = 810), AoU (United States; *n* = 559), BioMe (United States; *n* = 2,170), MGBB (United States; *n* = 2,141), UCLA-ATLAS (United States; *n* = 1,445), QBB (Qatar; *n* = 2,383) and BBSS (Poland, *N* = 355). The biobank studies include samples from (hospital) biobanks, prospective epidemiological and disease-based cohorts. Follow-up covers a total of 20 years with the earliest study starting follow-up in 2004 (AoU) and the latest study ending follow-up in 2024 (BioMe). Below we provide a detailed description of the population selected and ancestry definitions in each study.

### Inclusion and exclusion criteria for GLP1-RA analysis

For our GLP1-RA analysis we implemented the following inclusion and exclusion criteria. We required an initiation of a GLP1-RA treatment (ATC codes: A10BJ*, A10BX04, A10BX10, A10BX13, A10BX14) defined as the date of first medication purchase or medication prescription of GLP1-RA. Only first-time (GLP1-RA naive) users in the datasets were included. Individuals were required to be on a GLP1-RA treatment for at least 12 months. This was defined through prescription length, regular drug prescription refills or drug purchases, depending on the dataset and healthcare setting.

Individuals aged under 18 were excluded from the analysis. Furthermore, individuals were required to have at least one body weight measurement a maximum of 1 year before treatment initiation or within 14 days after initiation, if no measure before initiation was available. If we observed multiple weight measurements in this time frame, only the one closest to initiation was considered. This measurement defined our baseline weight variable. At least one body weight measurement between 26 and 52 weeks was necessary for each individual to be included in the analysis. Individuals were excluded if they underwent BS before or during the first year of GLP1-RA treatment.

### Inclusion and exclusion criteria for BS analysis

For our BS analysis we implemented the following inclusion and exclusion criteria. Individuals were included if they underwent any type of BS. The respected code definitions for BS varied across countries and healthcare systems and can be found in Supplementary Table 22. The proportions of all procedures across cohorts can be found in Supplementary Table 23. Individuals aged under 18 years at baseline were excluded from the analysis. Analogously to the GLP1-RA analysis, individuals were required to have at least one body weight measurement a maximum of 1 year before surgery or within 14 days after it, if no measure before baseline was available. If we observed multiple weight measurements in this time frame, only the one closest to initiation was considered. This measurement defined our baseline weight variable. In addition, at least one body weight measurement between 26 and 208 weeks was necessary for each individual to be included in the analysis.

### Outcome definitions

In our primary model, for both GLP1-RA and BS we defined the outcome to be the percentage change in body weight from baseline. The baseline body weight measured closest to *T*_0_ and between −52 weeks and +2 weeks from *T*_0_ was defined as *W*_0_. The second body weight (*mW*_1_) was defined as the median of body weight measurements between 26 and 52 weeks from *T*_0_ for the GLP1-RA analysis and between 180 and 1,460 days for the BS analysis. This was to enhance robustness against outlier measurements occurring in these intervals.

Therefore, the percentage change in body weight from baseline was calculated using

$$\% {\mathrm{Weight}}\; {\mathrm{change}}=\frac{m{W}_{1}\,-\,{W}_{0}}{{W}_{0}}\times 100.$$

In our secondary model, we used *mW*_1_ as an outcome.

### Genetic exposures

We identified 15 genetic variants (SNPs) and two PGS of interest for our analysis. Among the included SNPs, three are functionally characterized *GLP1R* variants (rs10305492, rs146868158, rs6923761) associated with random glucose levels that have shown a decreased or increased response to different endogenous and exogenous GLP1-RA^45^. Seven SNPs are *GLP1R* missense variants (rs1042044, rs10305420, rs3765467, rs10305421, rs2295006, rs10305510, rs201672448) with a frequency >1% in at least one ancestry group in gnomAD (v.4.1)^44^. One SNP is the only robustly replicated lead genome-wide significant variant (rs429358, *ApoE*) in a genome-wide association study of BMI change over time^41,61^. Two SNPs are *PCSK1* missense variants (rs6232, rs6235) that have been found associated with BMI variations^46^. And two SNPs (rs728996, rs17702901) were associated with an effect on excess BMI loss among BS patients^36^. A list of all genetic exposures and their frequencies across all major continental ancestry groups can be found in Supplementary Table 4. The included PGS are for BMI and T2D^62^. The PGS weights were taken from ref. ^62^ and obtained from UK Biobank. The scores were computed in each study using PLINK 2.0 (ref. ^63^). Because the PGS scores were derived from UK Biobank, we could not use the same score in UK Biobank, instead we used ref. ^64^.

### Statistical model

We used a linear regression model to investigate the effect of the chosen genetic exposures on weight change after initiation of GLP1-RA treatment and BS, respectively. In the primary model we adjusted for the baseline weight, sex, age at initiation of treatment or surgery in years, the first 20 genetic principal components (PCs) and study-specific covariates, such as the genotyping batch. In the GLP1-RA analysis we additionally adjusted for the medication type in the drug class.

We defined % weight change as the outcome variable in the primary model.

Primary model:

$$\begin{array}{l}\% {\mathrm{Weight}}\; {\mathrm{change}}={\beta }_{0}+{\beta }_{1}\times {\mathrm{Genetic}}\; {\mathrm{exposure}}+{\beta }_{2}\times {W}_{0}+{\beta }_{3}\times {\mathrm{Sex}}\\\qquad\qquad\qquad\qquad+{\beta }_{4}\times {\mathrm{Age}}\; {\mathrm{at}}\; {\mathrm{initiation}}+{\beta }_5 \times {\mathrm{Medication}}\;(\mathrm{only}\; \mathrm{for}\; \mathrm{GLP}1)\\\qquad\qquad\qquad\qquad+\mathop{\sum }\limits_{k\,=\,6}^{25}{\beta }_{k}\times {PC}1:20\,+\mathop{\sum }\limits_{i=26}^{n}{\beta }_{i}\times {\mathrm{Study}}\;{\mathrm{specific}}\; {\mathrm{covariates}}\end{array}$$

### Meta-analyses

We conducted ancestry-specific and multiancestry fixed-effect meta-analyses for each of the genetic exposures. Effect sizes were combined using fixed-effect inverse-variance weighting, as implemented in the R package meta^65^. For each exposure we reported the unadjusted *P* values, but considered as threshold for statistical significance *P* < 0.004 for GLP1-RA (that is, *P* < $\frac{0.05}{13}$, Bonferroni correction for 13 exposures tested) and *P* < 0.01 for BS (that is, *P* < $\frac{0.05}{5}$, Bonferroni correction for 5 exposures tested). We tested for heterogeneity in effect sizes between ancestries using Cochran’s *Q*-test^66^ and by inspecting the *I*^2^ statistics^67^, which is defined as:

$${I}^{2}=\left(\frac{Q-\mathrm{d.f.}}{Q}\right)\times 100 \%$$

where d.f. is equal to the number of studies − 1.

### Power calculation

To calculate the statistical power for the PGS effect sizes, we used a *t*-test for linear regression coefficient as implemented in the R package pwrs^68^ assuming a standard deviation for the outcome variable s.d.y = 7.8 (weighted combine s.d. across studies), a sample size of *n* = 6,750, a total number of predictors *k* = 25 and an adjusted *r*^2^ = 0.05 (coefficient of determination), to calculate power at *P* < 0.05 significance level.

For the coefficients of association with single variants, we estimated statistical power via the noncentrality parameter of the chi-squared distribution. We defined the noncentrality parameter as 2*f* (1 − *f*) *n* *β*^2^ where *f* is MAF, *n* is the effective sample size and *β* is the expected effect size^69^. We set a beta value of 0.3 (percent weight change from baseline) as the expected genetic effect on GLP1 response, based on findings from a recent large-scale study on genetic influences on drug response^52^.

### Cohort descriptions and ancestry definitions

#### All of Us

The AoU Research Program is a longitudinal cohort study aiming to enroll at least one million individuals across a diverse population in the United States. More than 245,000 individuals have genotype data. The sensitivity for single nucleotide variants was >98.7% and the precision was >99.9%.

Ancestry categories were based on gnomAD and inferred using principal components analysis (PCA) data. The patterns of ancestry and admixture were compared with self-identified race and ethnicity, and continuous ancestry inference using genome-wide genotypes resulted in concordant estimates. We restricted participants to an unrelated subset. The participants were further filtered based on the criteria described in the analysis plan (for example, $\frac{\mathrm{Per}\; \mathrm{ancestry}\; \mathrm{minimum}\; \mathrm{allele}\; \mathrm{count}}{\mathrm{Sample}\; \mathrm{size}}$ after filtering on availability of weight measurements and prescription and/or BS).

#### Bialystok Bariatric Surgery Study

The BBSS is a prospective cohort study of patients undergoing BS at the First Clinical Department of General and Endocrine Surgery at the Medical University of Bialystok. This is the primary receiving center for patients referred for BS in the province of Podlaskie Voivodeship and the largest center by number of bariatric surgeries performed in northeastern Poland. This center specializes in several bariatric surgical techniques including RYGB, gastric banding and SG. For this study, we selected only patients who underwent SG because it represents the vast majority (>90%) of all interventions performed at the center and to eliminate confounding variation in surgical technique. The BBSS began in 2015 and consisted of a battery of baseline tests established one month before the intervention and repeated at 1-, 3-, 6- and 12-month follow-up clinical visits. Subsequently, patients are examined every year after the first year. At each visit, all subjects underwent physical examination, body composition analysis and blood testing, as well as completed diet and physical activity questionnaires. All subjects give their informed consent for inclusion before participating in the study. The study is conducted in accordance with the Declaration of Helsinki, and the protocol was approved by the Ethics Committee of the Medical University of Bialystok (Project identification code: R-I-002/546/2015).

Genetic ancestry in the BBSS cohort was determined using a combination of self-reported race or ethnicity and genotype-based analysis. Self-reported race or ethnicity information was collected during baseline assessments. Additionally, genetic ancestry was further refined through genotype clustering analyses using reference populations from the 1000 Genomes Project.

#### BioMe

The BioMe Biobank, founded in 2007, is an ongoing EHR-linked biorepository that enrolls participants nonselectively from across the Mountain Sinai Health System. More than 60,000 participants have been recruited from >26 outpatients sites located in Manhattan and Queens, and recruitment is ongoing. We restricted participants to an unrelated subset (based on second-degree or greater KING-derived kinship coefficients) who had been genotyped with either the Global Screening Array or Global Diversity Array. We then filtered this population to those adhering to the criteria described in the analysis plan (for example, $\frac{\mathrm{Per}\;\mathrm{ancestry}\;\mathrm{minimum}\;\mathrm{allele}\;\mathrm{count}}{\mathrm{Sample}\;\mathrm{size}}$ after filtering on availability of weight measurements and prescription and/or BS).

Genetically determined ancestry group based on ADMIXTURE with *K* = 10 (where *K* refers to the number of ancestral clusters inferred in the ADMIXTURE analysis), with grouping based on which 1000 Genomes phase 3 reference samples (with superpopulation labels as reference) showed the highest proportion for a given class. The choice for *K* = 10 was based on a combination of minimizing cross-validation errors for admixture analysis, as well as minimizing the Frobenius distance of each ADMIXTURE matrix *K* with the matrix outputted by Genetic Relationship and Fingerprinting.

#### Estonian Biobank

ESTBB is a population-based biobank that involves more than 212,000 adult participants (about 20% of the Estonian adult population). The Estonian Biobank Project was initiated in 1999; data collection began in 2002. Initially, participants were recruited by general practitioners across Estonia from among individuals visiting general practitioners’ offices and hospitals. Consenting adults donated blood samples for DNA extraction, underwent anthropometric, blood pressure and resting heart rate measurement, and provided extensive information on demographic characteristics, genealogy, education, occupation and lifestyle, as well as health status and medical history^70^. From 2017 onward, the process of joining ESTBB was simplified: new joiners were required to sign an online consent form, visit a healthcare provider or pharmacy to provide a blood sample, and fill in an online questionnaire. Additional data are collected through linkage with various national databases and registries and through subsequent studies involving different subsets of participants.

Participants were genotyped using the Illumina Global Screening Array v.1.0, v.2.0 and v.2.0_EST. Samples were genotyped and PLINK format files created using Illumina GenomeStudio v.2.0.4. Individuals with call rates below 95% or whose sex in phenotype data did not match X chromosome heterozygosity were excluded. Before imputation, variants were filtered by call rate <95%, Hardy–Weinberg equilibrium *P* < 1 × 10^−4^ (autosomal variants only) and MAF < 1%. A population-specific imputation reference panel of 2,297 whole-genome sequencing (WGS) samples was used^71^.

We aligned participants to 21 major ancestry groups similarly to Privé^72^. We removed participants with nonEuropean assigned group ancestry, keeping Europeans, Finns and Italians.

#### Helsinki University Hospital/Helsinki Biobank

The Helsingin Biopankki (Helsinki Biobank) was founded in 2015 by HUS Helsinki University Hospital, University of Helsinki, the Joint Municipality for Health and Social Services in the Kymenlaakso Valley (Kymsote) and the South Karelia Social and Health Care District (Exote), and is the largest hospital biobank in Finland. Approximately 80,000 patients have given a blood sample based on biobank consent and used for research purposes. Diagnostic formalin-fixed paraffin-embedded tissue samples from approximately one million patients have been transferred to the biobank’s sample collection. The biobank’s samples were combined with clinical patient data from the hospital’s database (HUS Datapool) in accordance with the description in the research plan recommended by the ethics committee. Data that are run together can usually include the sampling date, the donor’s gender, histological and cytological examinations, diagnoses, care measures and laboratory examination results.

#### Mass General Brigham Biobank

Mass General Brigham is an integrated healthcare system located in Boston, Massachusetts for more than 1.5 million individuals per year. The MGBB has recruited more than 140,000 participants since 2008. More than 65,000 participants have genotype data sequenced using the Illumina Multi-Ethnic Genotyping assay or the Illumina Global Screening Array. All genotyped biospecimens had at least 99% call rate per array and were confirmed for sex concordance using sex in EHR and sex computed from array data.

Ancestry was defined using high-dimensional PCs, specifically top 30 genetic PCs and a network-based clustering approach. We restricted participants to an unrelated subset. The participants were further filtered based on the criteria described in the analysis plan (for example, $\frac{\mathrm{Per}\;\mathrm{ancestry}\;\mathrm{minimum}\;\mathrm{allele}\;\mathrm{count}}{\mathrm{Sample}\;\mathrm{size}}$ after filtering on availability of weight measurements and prescription and/or BS).

#### UCLA-ATLAS (Atlas Biobank)

ATLAS is an EHR-linked biobank across multiple institutions in the UCLA Health System. After participants signed informed consent for participation to the UCLA-ATLAS Community Health Initiative, biological samples in the form of de-identified blood samples were collected and subsequently processed for DNA extraction and genotyping. The data analyzed in this manuscript draw from a ‘frozen snapshot’ of ATLAS data encompassing all available samples up to 15 February 2024, totaling *n* = 53,829. The corresponding EHR data for ATLAS participants were sourced from the UCLA Data Discovery Repository. This repository operates under the auspices of the UCLA Health Office of Health Informatics Analytics and the UCLA Institute of Precision Health. Patient Recruitment and Sample Collection for Precision Health Activities at UCLA is an approved study by the UCLA Institutional Review Board. Extensive details on ATLAS genotyping and quality control were previously reported in ref. ^73^. In short, participants in the ATLAS initiative were genotyped using a custom genotyping array constructed from the Global Screening Array with the Infinium Global Screening Array-24 Kit (Illumina), a multidisease drop-in panel aligned with the GRCh38 assembly. SNPs were subjected to removal if they were unmapped, strand-ambiguous, duplicates or exhibited >5% missingness. Samples with missingness exceeding >5% were excluded, as were duplicates (including identical twins or triplets) with preference given to individuals with the lowest missing rate in each pair. Imputation to the TOPMedFreeze5 was conducted using the Michigan Imputation Server^74^. After imputation, SNPs with a quality score (*R*^2^) below 0.90 were excluded, for PGS calculation SNPs with a MAF below 0.1% were filtered out.

The genetic ancestry of ATLAS individuals was estimated by assessing their proximity to 1000 Genome super populations on the PC space. We computed the top 20 PCs using bigsnpr R software with default parameters. Subsequently, leveraging the superpopulation label and PCs of the 1000 Genome individuals, we train a *K*-nearest neighbors model to assign genetic ancestry labels to each ATLAS individual. To optimize model performance, we used tenfold cross-validation to select the optimal hyperparameter (*k*) from a range of values (*k* = 5, 10, 15, 20). In instances in which an individual was assigned to multiple ancestries with probability >0.5 or was not assigned to any cluster, their ancestry was labeled as unknown. We clustered 94% of the ATLAS participants into one of the five 1000 Genome superpopulation (African (AFR), admixed American (AMR), East Asian (EAS), European (EUR), Middle Eastern (MID), South Asian (SAS)) while the remaining individuals’ ancestry could not be ascertained.

#### UK Biobank

The UKBB is a large-scale biomedical database and research resource containing in-depth genetic and health information from half a million UK participants. Upon signing informed consent, participants provided biological samples, including de-identified blood samples, which were processed for DNA extraction and genotyping. The dataset analyzed in this manuscript is derived from a ‘frozen snapshot’ of the UKBB data, encompassing all available samples up to 31 January 2024, totaling *n* = 502,536. Corresponding EHR data for UKBB participants were sourced from the UK Biobank Resource, which operates under the auspices of the UK Biobank Steering Committee and has received ethical approval from the North West Multi-centre Research Ethics Committee. Detailed information on UKBB genotyping and quality control has been previously reported^75^. Samples were genotyped at the Affymetrix Research Services Laboratory in Santa Clara, California, USA. Upon receipt of a 96-well plate containing 94 UK Biobank samples, Affymetrix added two control individuals (from 1000 Genomes) to the same well positions on each plate. Genotypes were then called from the resulting intensities in batches of ~4,700 samples (~4,800 including the controls) using Affymetrix Power Tools software and Affymetrix Best Practices Workflow. After genotype calling, Affymetrix performed quality control in each batch separately, to exclude SNPs with poor cluster properties. If an SNP did not meet the Affymetrix prescribed QC thresholds in a given batch, it was set to missing in all individuals from that batch. Affymetrix also checked sample quality (such as DNA concentration) and genotype calls were provided only for samples with sufficient DNA metrics.

The ancestry composition of UKBB participants was defined based on self-reported ethnicities and genetic PCA. Participants self-reported their ethnic backgrounds into categories such as White, Mixed, Asian or Asian British, Black or Black British, and Other ethnic groups, with the majority (~94%) identifying as White British. Genetic PCA was used to capture the underlying population structure and define genetic ancestry more precisely, categorizing participants into groups such as European, South Asian, African, East Asian and other ancestries. This approach ensured a nuanced understanding of the genetic and demographic diversity in the cohort, enabling robust and reliable biomedical research.

To capture population structure specific to the UKBB cohort, we performed principal component analysis of ~150,000 UKBB samples using ~100,000 SNPs. These PCs can be used to identify samples with similar ancestry or to control for population structure in association studies.

#### Qatar Biobank

This study was based on the second release of 14,060 Qatari participants (all from the same ancestry, MID) from the population-based Qatar Genome Program (QGP). Ethical approval was provided by the institutional review board of the QBB (Protocol no. QF-QBB-RES-ACC-00205). A detailed description of the recruitment process and collection of phenotypic data by the QGP and the QBB was previously reported^76^. A signed consent was obtained from all included participants by the QBB, and WGS data used in the current study were obtained from the QGP as previously described^77^. All samples were sequenced on Illumina HiSeq X instruments to obtain WGS with a target average depth of coverage of 30×. The read mapping, variant calling and joint variant calling were performed using Sentieon’s DNASeq pipeline v.201808.03, following the BWA-GATK Best Practice Workflow and using the GRCh38/hg38 reference genome. The produced gVCF were jointly called to produce one multisample VCF file for the whole cohort. Clinical data are extracted from the EMR of the Hamad Medical Corporation.

### Reporting summary

Further information on research design is available in the Nature Portfolio Reporting Summary linked to this article.

## Online content

Any methods, additional references, Nature Portfolio reporting summaries, source data, extended data, supplementary information, acknowledgements, peer review information; details of author contributions and competing interests; and statements of data and code availability are available at 10.1038/s41591-025-03645-3.

## Supplementary information

Supplementary Information Supplementary Methods, Results and Figs. 1–12.

Reporting Summary

Supplementary Tables 1–23.

## Source data

Source Data Fig. 1, 2a, 2b and Extended Data Fig. 2–4. An Excel workbook containing the source data to generate Figs 1, 2a, 2b and Extended Data Figs 2–4.

## Data Availability

The Helsinki Biobank can provide access for research projects within the scope regulated by the Finnish Biobank Act, which is research utilizing the biobank samples or data for the purposes of promoting health, understanding the mechanisms of disease or developing products and treatment practices used in health and medical care. De-identified data of the MGBB that supports this study is available from the MGB Biobankportal at https://www.massgeneralbrigham.org/en/research-and-innovation/participate-in-research/biobank/for-researchers. Restrictions apply to the availability of these data, which are available to MGB-affiliated researchers via a formal application. UK Biobank data are available through a procedure described at http://www.ukbiobank.ac.uk/using-the-resource/. Registered researchers whose institutions have Data Use and Registration Agreements in place with All of Us that include the Controlled Tier can access genomic data. Clinical and genotype data from UCLA ATLAS Community Health Initiative patients, de-identified for research purposes, are accessible to UCLA-approved researchers through the Discovery Data Repository (DDR). BioMe data is available through a process described at https://icahn.mssm.edu/research/ipm/programs/biome-biobank/researcher-faqs. Pseudonymized data and/or biological samples can be accessed for research and development purposes in accordance with the Estonian Human Genome Research Act. To access data, the research proposal must be approved by the Scientific Advisory Committee of the Estonian Biobank as well as by the Estonian Committee on Bioethics and Human Research. For more details on data access and relevant documents, please see https://genomics.ut.ee/en/content/estonian-biobank#dataaccess. For access to the Bialystok Bariatric Surgery Study please refer to the Medical University of Bialystok. The data and biosamples collected or generated by QBB will be made available to researchers employed within or otherwise contractually bound to public and private institutions that conduct scientific research and that meet the requirements detailed in the Qatar Biobank Research Access policy. The PGS weights used in this study are available in refs. ^62^ and ^64^. Source data are provided with this paper.

## Code availability

The study utilized previously published analysis tools as described in the Methods. Additional code used for these analyses is available at https://github.com/dsgelab/glp1-bs-genetics/.
