# Supplementary material for: Association between plausible genetic factors and weight loss from GLP1-RA and bariatric surgery
Source: Nat Med. 2025 Apr 18;31(7):2269–76. doi: 10.1038/s41591-025-03645-3 (PMC12283387; doi:10.1038/s41591-025-03645-3)
Supplement: Supplementary file 1 — Supplementary Methods, Results and Figs. 1–12. [file 41591_2025_3645_MOESM1_ESM.pdf]

nature medicine

Supplementary information

https://doi.org/10.1038/s41591-025-03645-3

Association between plausible genetic factors and weight loss from GLP1-RA and bariatric surgery

In the format provided by the authors and unedited

Supplementary Material – Sensitivity analyses

All sensitivity analyses focused on the effects of the two polygenic scores (BMI and T2D PGS) rather than single genetic variants. Although the primary analyses had sufficient statistical power to detect effects from individual variants, no significant associations were observed. Consequently, we prioritized the polygenic scores in the sensitivity analyses, as they capture aggregated genetic effects and provide a more comprehensive evaluation of potential genetic contributions.

Supplementary Methods

Sensitivity analysis: Utilizing the minimum weight (minW1) instead of the median weight (mW1)

To explore the impact of different approaches to defining postoperative or post-drug therapy weight measurements, we conducted a sensitivity analysis using the minimum observed weight (minW1) instead of the median weight (mW1) as the primary outcome. This analysis aimed to evaluate whether our choice of using the median weight, which provides a more conservative and robust estimate by minimizing the influence of outliers, influenced the observed results.
The modified outcome variable was defined as:

$$\%\, weight\ change\ =\ \frac{minW_1 - W_0}{W_0} * 100$$

This sensitivity analysis was applied to both the GLP1-RA and BS cohorts.

The results of this analysis are reported in **Supplementary Tables 9-10, Supplementary Figures 1-2** and further discussed in the **Supplementary Results**.

Sensitivity analysis: Shorter follow-up (12 months) for the BS analysis

To assess whether the observed effects in the bariatric surgery (BS) analysis were influenced by the longer follow-up periods typically associated with this intervention, we conducted a sensitivity analysis limiting the follow-up period to 12 months post-surgery. This shorter follow-up

period aligns with the timeframe used in the GLP1-RA analysis, allowing for a more direct comparison of weight loss dynamics between the two treatment groups.

For this analysis, only weight measurements within the first 12 months post-surgery were considered. This sensitivity analysis included a total of 3,711 individuals, categorized by genetic ancestry as follows: AFR - 461; AMR - 124; EUR - 1979; MID - 1147.

The results of this analysis are reported in **Supplementary Table 11, Supplementary Figures 3-4** and further discussed in the **Supplementary Results**.

Sensitivity analysis: Adjusting for T2D status in the statistical model

To evaluate whether a diagnosis of type 2 diabetes (T2D) independently affects weight loss outcomes and to explore the robustness of our findings when accounting for T2D, we conducted a sensitivity analysis. In this analysis, T2D status was included as an additional covariate in the statistical model alongside the covariates used in the primary analysis. This adjustment allowed us to assess the potential contribution of T2D to weight loss variability and ensure that our findings were not biased by the exclusion of this factor.

The modified statistical model was defined as follows:

$$\%\, weight\ change \ = \beta_0 + \beta_1 * genetic\ exposure \ + \beta_2 * W_0 + \beta_3 * sex + \beta_4 * age\ at\ initiation \ + \beta_5 * medication\ (only\ for\ GLP1) \ + \beta_6 * T2D\ status \ + \sum_{k=7}^{26} \beta_k * PC1{:}\,20 + \sum_{i=27}^{n} \beta_i * study\ specific\ covariates$$

This sensitivity analysis was applied to both the GLP1-RA and BS cohorts.

The results of this analysis are reported in **Supplementary Table 12-13, Supplementary Figure 5** and further discussed in the **Supplementary Results**.

Sensitivity analysis: Including only liraglutide/semaglutide in GLP1-RA analysis

To explore whether the genetic and weight loss associations differ when focusing solely on the GLP1-RAs approved for obesity treatment, we conducted a sensitivity analysis including only patients treated with liraglutide or semaglutide. These medications have demonstrated greater efficacy for weight loss compared to other GLP1-RAs and are next to a T2D indication approved for obesity treatment.

Patients in this analysis may have been prescribed liraglutide or semaglutide for either T2D or obesity, as both indications are common. We did not restrict the analysis to only patients with obesity to ensure sufficient sample size and statistical power, as including patients with T2D broadened the cohort and extended the treatment time horizon.

This sensitivity analysis included a total of 4,431 individuals, categorized by genetic ancestry as follows: AFR - 635; AMR - 505; EAS - 769; EUR - 1670; MID - 701; SAS - 51.

The results of this analysis are reported in **Supplementary Table 14, Supplementary Figures 6-7** and further discussed in the **Supplementary Results**.

Sensitivity analysis: Stricter inclusion criteria for the BS analysis

To address the potential influence of preoperative low-calorie meal replacement diets on weight measurements, we conducted a sensitivity analysis with stricter inclusion criteria for the BS cohort. This analysis included only participants with weight measurements taken at least 30 days prior to surgery, within the time window of -365 days to -30 days relative to the day of surgery.

The Polish cohort (BBSS) was excluded from this analysis, as weight measurements for this cohort were only available on the day of surgery. This adjustment aimed to minimize the potential confounding effects of pre-surgical dietary interventions on baseline weight measurements and subsequent weight change calculations.

This sensitivity analysis included a total of 3,710 individuals categorized by genetic ancestry as follows: AFR - 510; AMR - 140; EUR - 1913; MID - 1147.

The results of this analysis are reported in **Supplementary Table 15, Supplementary Figures 8-9** and further discussed in the **Supplementary Results**.

Sensitivity analysis: Stratifying BS analysis by RYGB and SG procedures

The breakdown of surgery procedures across cohorts revealed that the vast majority of individuals underwent either Roux-en-Y gastric bypass (RYGB, 52.3% on average) or sleeve gastrectomy (SG, 43.6% on average; **Supplementary Table 23**). This distribution provided the rationale for conducting a sensitivity analysis stratified by these two procedures, allowing us to assess whether the observed effects varied by surgery type.

This stratified analysis included a total of 4,036 individuals categorized by genetic ancestry as follows: AFR - 492; AMR - 133; EUR - 2287; MID - 1124.

The results of this analysis are reported in **Supplementary Tables 16-17, Supplementary Figures 10-11** and further discussed in the **Supplementary Results**.

Sensitivity analysis: Model using post-treatment weight as outcome and including interaction between baseline weight and each genetic exposure

As previous works have argued that using percentage changes in weight loss pre vs post therapy may be statistically inappropriate and inefficient [PMID: 11459516, 24022766], we considered a model using post-treatment weight as outcome, including the same predictors as in the main model and an additional term modeling the interaction between baseline weight and each genetic exposure.

The modified statistical model was defined as follows:

$mW_1 = \beta_0 + \beta_1 * genetic\ exposure + \beta_2 * W_0 + \beta_3 * genetic\ exposure * W_0 +$

$$\beta_4 * sex + \beta_5 * age\ at\ initiation\ + \beta_6 * medication\ (only\ for\ GLP1) + \sum_{k=7}^{26} \beta_k * PC1{:}\ 20\ +$$

$$\sum_{i=27}^{n} \beta_i * study\ specific\ covariates$$

The results of this analysis are reported in **Supplementary Tables 18-19** and further discussed in the **Supplementary Results**.

Sensitivity analysis: Model without adjustment for baseline weight (W0)

In our primary analysis, we adjusted for baseline weight to isolate the genetic effect on post-treatment weight, aiming to capture the total genetic contribution. However, it is also relevant to consider the total causal genetic effect specifically on the weight change induced by the treatment.

It has further been proposed that adjusting for a baseline variable, such as baseline weight (W0), which is influenced by the same genetic factors as the outcome variable, may introduce bias and lead to spurious association, introducing the baseline genetic effect on the measurement in the estimation of the pharmacogenetic effect [PMID: 38633781]. In general, it is hypothesized that adjusting for a baseline measure in observational change analysis might introduce a backdoor path, allowing information to flow between the exposure (the genetics) and the outcome (the change) through an unintended path separate from the one created by the treatment in our case, this potentially opens bias inducing spurious associations between the exposure and the outcome [PMID: 25401453, 15987729]. Nonetheless, the magnitude of the potential bias with respect to the actual presence or absence of an effect of the exposure is still unclear. To evaluate whether the inclusion of baseline weight in our model might have affected the observed genetic associations, we conducted a sensitivity analysis in which W0 was excluded from the statistical model.

The modified statistical model was defined as follows:

$$\%\ weight\ change\ = \beta_0 + \beta_1 * genetic\ exposure + \beta_2 * sex + \beta_3 * age\ at\ initiation\ +$$

$$\beta_4 * medication\ (only\ for\ GLP1) + \sum_{k=5}^{24} \beta_k * PC1{:}\ 20\ + \sum_{i=25}^{n} \beta_i * study\ specific\ covariates$$

This sensitivity analysis was applied to both the GLP1-RA and BS cohorts.

The results of this analysis are reported in **Supplementary Table 20-21, Supplementary Figure 12** and further discussed in the **Supplementary Results**.

Supplementary Results

Sensitivity analysis: Utilizing the minimum weight (minW1) instead of the median weight (mW1)

Using the minimum weight (minW1) as the primary outcome resulted in a higher observed weight loss compared to the median weight (mW1), consistent with expectations.

For the GLP1-RA cohort, the average body weight change across studies was -6.00% (ranging from -4.10% to -11.75%) (**Supplementary Figure 1**). As in the primary analysis, no significant genetic effects explaining the heterogeneity in weight loss outcomes were observed in this cohort (**Supplementary Table 9** and **Supplementary Figure 2**).

For the BS cohort, the average body weight change was -25.25% (ranging from -17.77% to -39.50%) (**Supplementary Figure 1**). The previously observed effect of the BMI PGS on weight change was confirmed, albeit with a lower effect estimate ($\beta_{BMI\ PGS}$ = 0.39 % weight change compared to baseline for 1 SD change in the polygenic score, $P = 1.51 \times 10^{-2}$) (**Supplementary Table 10** and **Supplementary Figure 2**).

These findings demonstrate that the choice of postoperative or post-treatment weight definition does not alter the conclusions of our primary analysis. The genetic effect of the BMI PGS remains evident in the context of BS but is not observed in GLP1-RA users, consistent with our main findings.

This sensitivity analysis underscores the robustness of our results and strengthens our conclusion that genetic effects were detected only in the BS cohort for explaining weight loss variability.

Sensitivity analysis: Shorter follow-up (12 months) for the BS analysis

Limiting the follow-up period for BS to 12 months yielded an average body weight change of -21.72% (ranging from -16.43% to -29.60%), which is consistent with our main findings of -21.17% average body weight change. This suggests that the majority of weight reduction occurs within the first year post-surgery (**Supplementary Figure 3**).

The effect of the BMI PGS on weight change during this shorter follow-up period was estimated at $\beta_{BMI\ PGS}$ = 0.43% weight change per 1 SD change in the polygenic score. While this result was nominally significant ($P = 2.69 \times 10^{-2}$), it did not reach statistical significance after correcting for multiple tests **(Supplementary Table 11** and **Supplementary Figure 4**).

These findings suggest that while the majority of weight loss occurs within the first year, the shorter follow-up does not fully capture the statistically significant genetic effect pulling towards higher weight observed in the primary analysis and other sensitivity analyses. This highlights the importance of the extended follow-up period used in the primary analysis to provide a more accurate representation of long-term outcomes and genetic contributions.

Sensitivity analysis: Adjusting for T2D status in the statistical model

This sensitivity analysis confirmed the previously observed effect of the BMI PGS on weight change following BS, with a comparable effect estimate to the primary analysis ($\beta_{BMI\ PGS}$ = 0.59% weight change compared to baseline per 1 SD change in the polygenic score, $P = 6.60 \times 10^{-4}$) (**Supplementary Table 13** and **Supplementary Figure 5**).

Among GLP1-RA users, no genetic effect explaining the heterogeneity in weight loss outcomes was observed, consistent with the findings from the primary analysis (**Supplementary Table 12** and **Supplementary Figure 5**).

The analysis further revealed a significant effect of T2D on weight change among GLP1-RA users (β_{T2D} = 0.54% weight change compared to baseline, $P = 1.90 \times 10^{-2}$), indicating that

individuals with T2D face greater difficulty in losing weight compared to those without T2D, aligning with existing literature (**Supplementary Table 12**). On the other hand, no significant effect of T2D was observed among individuals undergoing BS.

Sensitivity analysis: Including only liraglutide/semaglutide in GLP1-RA analysis

The sensitivity analysis revealed an average body weight change of -4.69% (ranging from -1.24% to -12.70%) among patients treated with liraglutide or semaglutide, which was larger than the -3.93% weight loss observed in our main analysis (**Supplementary Figure 16**). This finding aligns with the higher efficacy of these medications for weight loss compared to other GLP1-RAs.

Despite this increased weight loss, no significant genetic effects explaining the variability in weight loss outcomes were observed in this analysis, consistent with the results of the main analysis (**Supplementary Table 14** and **Supplementary Figure 7**).

Sensitivity analysis: Stricter inclusion criteria for the BS analysis

Limiting the inclusion criteria to participants with weight measurements available at least 30 days prior to surgery resulted in a slightly larger observed average body weight change of -23.64% (ranging from -18.54% to -29.50%) compared to the primary analysis, which reported an average weight change of -21.17% (ranging from -15.00% to -27.72%) (**Supplementary Figure 8**).

Importantly, this sensitivity analysis confirmed the previously observed effect of the BMI PGS on weight change following bariatric surgery. The effect size ($\beta_{BMI\ PGS}$ = 0.66% weight change compared to baseline per 1 SD change in the polygenic score, $P = 2.40 \times 10^{-4}$) remained comparable to that of the primary analysis (**Supplementary Table 15** and **Supplementary Figure 9**), which reported an effect size of $\beta_{BMI\ PGS}$ = 0.70%.

This finding supports the assumption that excluding weight measurements taken closer to the surgery date removes the influence of preoperative dietary interventions, which can cause substantial weight loss prior to surgery.

Sensitivity analysis: Stratifying BS analysis by RYGB and SG procedures

We observed that both surgery procedures had nearly identical average body weight changes: -22.30% (ranging from -14.90% to -31.60%; **Supplementary Figure 10**) for RYGB and -22.10% (ranging from -14.20% to -27.30%) for SG. For RYGB, we observed a significant effect of the BMI PGS on weight change (0.83% weight change compared to baseline per 1 SD change in the polygenic score, P = 1.49×10^{-4}), which aligns with and confirms the effect observed in our primary analysis (**Supplementary Table 16** and **Supplementary Figure 11**). However, no significant genetic effect was observed for SG (0.31% weight change compared to baseline per 1 SD change in the polygenic score, P = 2.11×10^{-1}; **Supplementary Table 17**).

Notably, the Qatari (QBB) cohort contained only 23 individuals who underwent SG, resulting in a sample size too small to calculate standard errors for the estimates in the model. Consequently, the QBB cohort, and by extension, the Middle Eastern (MID) ancestry group, did not contribute to the SG sub-analysis. Given the lack of separation between techniques and studies, it is difficult to discern whether the effects we observed are driven by the surgical procedures themselves, the cohort-specific characteristics, or ancestry-related factors. Therefore, we cannot draw definitive conclusions from this analysis.

Sensitivity analysis: Model using post-treatment weight as outcome and including interaction between baseline weight and each genetic exposure

Using the alternative model, we identified one missense variant in the *GLP1R* genes, *rs3765467*, for which we observed a statistically significant interaction with baseline weight ($\beta_{w0*rs3765467}$ = 0.09 kg weight change from baseline weight, $P = 2.7 \times 10^{-4}$) on the weight measured post GLP1-RA treatment after multiple testing correction. When considering nominal significance, another missense variant in the *GLP1R* genes, *rs6923761*, had a statistically

significant interaction with baseline weight ($\beta_{w0*rs6923761}$ = 0.02 kg weight change from baseline weight, P = 0.01) on the weight measured post GLP1-RA treatment (**Supplementary Table 18**).

As for the BS analysis, we only observed a nominally significant interaction of the BMI PGS with baseline weight ($\beta_{w0*BMI\ PGS}$ = 0.04 kg weight change from baseline weight, $P = 8.7 \times 10^{-3}$) on the post-operative weight after multiple testing correction (**Supplementary Table 19**).

Sensitivity analysis: Model without adjustment for baseline weight (W_0)

Removing baseline weight (W_0) from the statistical model led to a reduction in the estimated effect size of the BMI PGS in the BS analysis. The effect size was reduced to $\beta_{BMI\ PGS}$ = 0.15% weight change compared to baseline per 1 SD change in the polygenic score ($P = 4.20 \times 10^{-1}$), compared to $\beta_{BMI\ PGS}$ = 0.70% in the primary model. While the directionality of the effect remained consistent, the association was no longer statistically significant.

For the GLP1-RA analysis, no significant genetic effects were observed, consistent with the findings of the primary analysis.

These results demonstrate that adjusting for baseline weight yielded a statistically significant genetic effect in the primary BS analysis, which was attenuated when baseline weight was removed from the model. Further details can be found in **Supplementary Tables 20-21** and **Supplementary Figure 12**.

Supplementary Figure 1 – Sensitivity analysis – “utilizing the minimum weight (minW1) instead of the median weight (mW1)”: Average percentage change in body weight

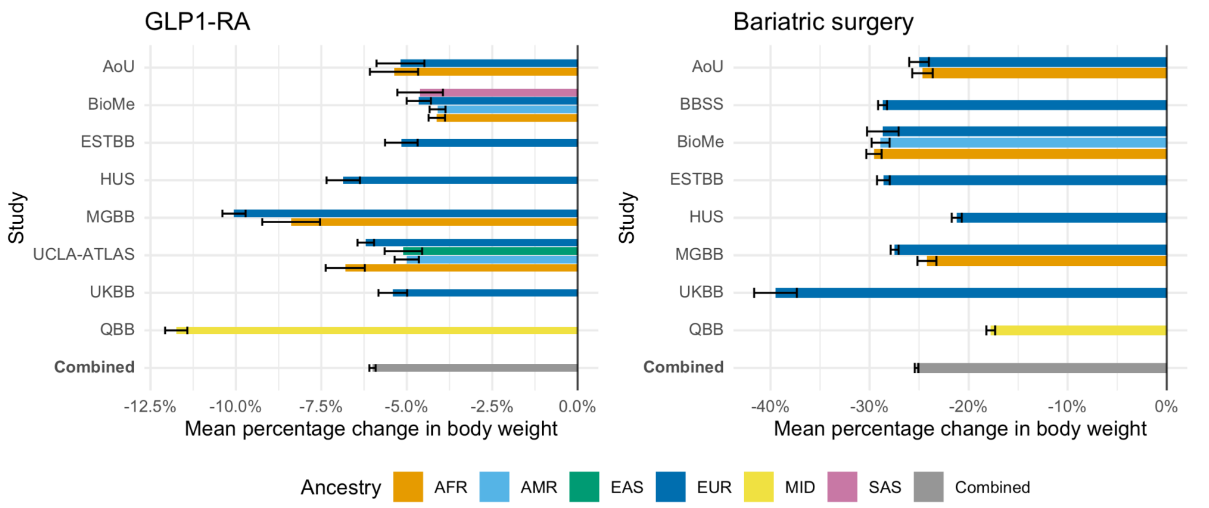

Supplementary Figure 1 | Sensitivity analysis – utilizing the minimum weight (minW1) instead of the median weight (mW1): Average percentage change in body weight for 6 750 GLP1-RA users and 4 210 bariatric surgery patients.

Bars represent the mean percentage change in body weight within each study and ancestry, and the overall combined weighted mean change. Error bars represent standard errors.

The average percentage change in body weight is calculated using the minimum postoperative or post-treatment weight, as per the relaxed outcome definition. Total N = 6 750, sample size per each cohort/ancestry can be found in **Table 1**.

The method for this sensitivity analysis is described in the **Supplementary Methods**.

AoU, All of Us; BBSS, Bialystok Bariatric Surgery Study; ESTBB, Estonian Biobank; HUS, Helsinki Biobank; MGBB, Mass General Brigham Biobank; UCLA-ATLAS, Atlas Biobank; UKBB, UK Biobank; QBB, Qatar Biobank; AFR, African; AMR, Admixed American; EAS, East Asian; EUR, European; MID, Middle Eastern; SAS, South Asian

Supplementary Figure 2 – Sensitivity analysis – "utilizing the minimum weight (minW1) instead of the median weight (mW1)": Effect of T2D and BMI polygenic scores on body weight changes

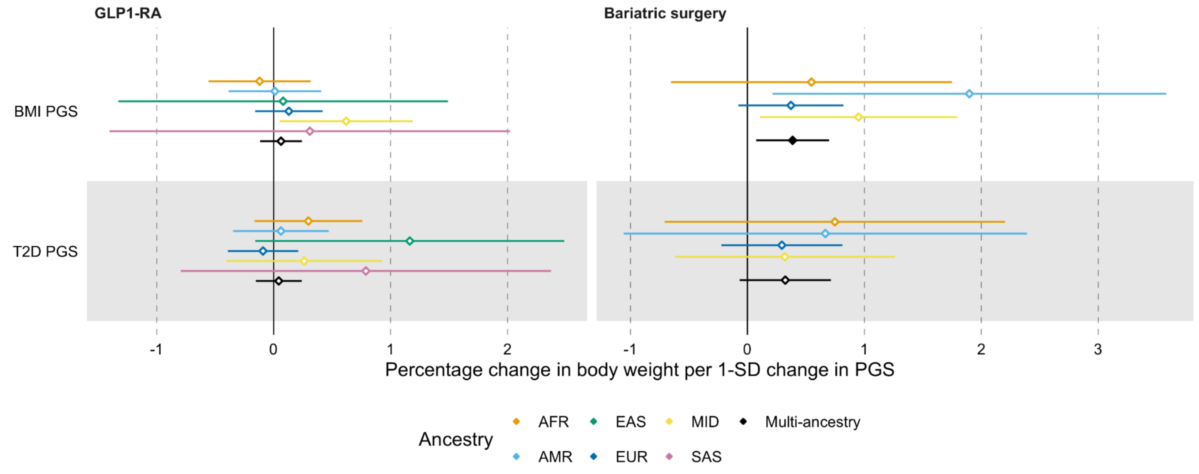

Supplementary Figure 2 | Sensitivity analysis – utilizing the minimum weight (minW1) instead of the median weight (mW1): Effect of T2D and BMI polygenic scores on body weight changes associated with GLP1-RA treatment and bariatric surgery.
Ancestry-specific and multi-ancestry meta-analysis effect sizes for association between percentage change in body weight and PGS for BMI and type 2 diabetes. Dots represent the percentage change in body weight per one standard deviation change in PGS, error bars represent the 95% confidence interval. For both panels, coefficients are estimated from a linear regression model, separately for each genetic exposure. Full dots represent statistical significance at $P < 0.025$ (Bonferroni adjusted for 2 independent tests). P values are two-sided and were calculated by dividing the coefficient values by their standard errors and observing the probability mass corresponding to equal or more extreme values from both tails of a t-student distribution. Exact P values can be found in the **Supplementary Tables**.
The average percentage change in body weight is calculated using the minimum postoperative or post-treatment weight, as per the relaxed outcome definition.
Total N = 6 750, sample size per each cohort/ancestry can be found in **Table 1**.
The method for this sensitivity analysis is described in the **Supplementary Methods**.
AFR, African; AMR, Admixed American; EAS, East Asian; EUR, European; MID, Middle Eastern; SAS, South Asian; PGS, Polygenic Score.

Supplementary Figure 3 – Sensitivity analysis – "shorter follow-up (12 months) for the BS analysis": Average percentage change in body weight

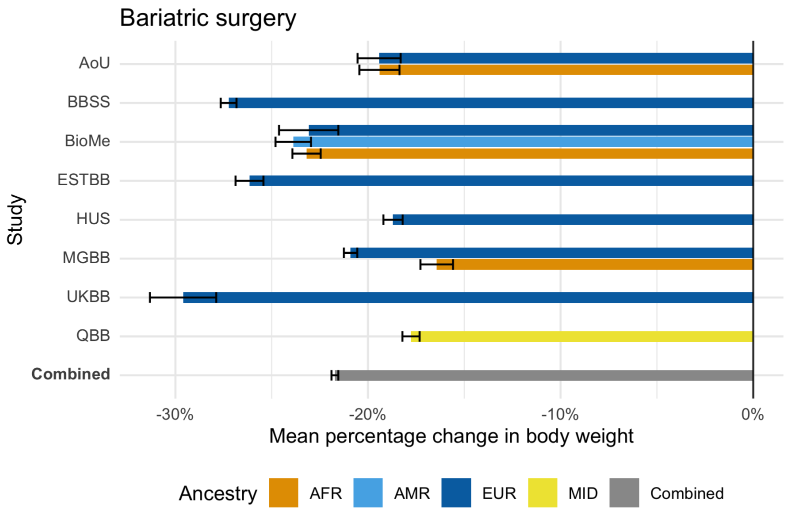

Supplementary Figure 3 | Sensitivity analysis – shorter follow-up (12 months) for the BS analysis: Average percentage change in body weight for 3 711 bariatric surgery patients.
Bars represent the mean percentage change in body weight within each study and ancestry, and the overall combined weighted mean change. Error bars represent standard errors.
Total N = 3 711, sample size per each cohort/ancestry can be found in the Supplementary Methods.
The method for this sensitivity analysis is described in the **Supplementary Methods**.
AoU, All of Us; BBSS, Bialystok Bariatric Surgery Study; ESTBB, Estonian Biobank; HUS, Helsinki Biobank; MGBB, Mass General Brigham Biobank; UKBB, UK Biobank; QBB, Qatar Biobank; AFR, African; AMR, Admixed American; EAS, East Asian; EUR, European; MID, Middle Eastern; SAS, South Asian

Supplementary Figure 4 – Sensitivity analysis – "shorter follow-up (12 months) for the BS analysis": Effect of T2D and BMI polygenic scores on body weight changes

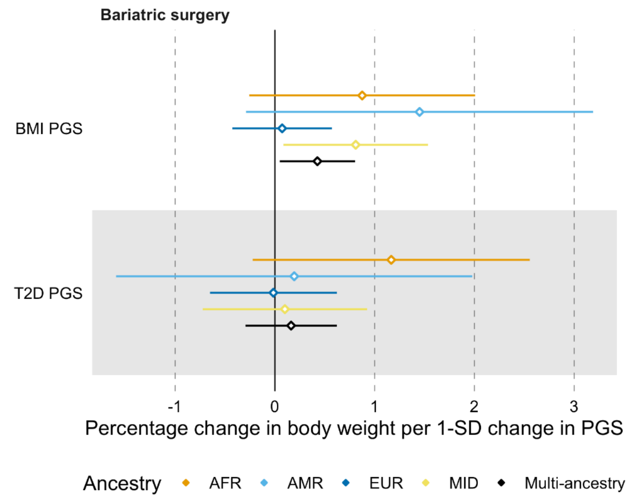

Supplementary Figure 4 | Sensitivity analysis – shorter follow-up (12 months) for the BS analysis: Effect of T2D and BMI polygenic scores on body weight changes associated with GLP1-RA treatment and bariatric surgery.
Ancestry-specific and multi-ancestry meta-analysis effect sizes for association between percentage change in body weight and PGS for BMI and type 2 diabetes. Dots represent the percentage change in body weight per one standard deviation change in PGS, error bars represent the 95% confidence interval. Coefficients are estimated from a linear regression model, separately for each genetic exposure. Full dots represent statistical significance at P < 0.025 (Bonferroni adjusted for 2 independent tests). P values are two-sided and were calculated by dividing the coefficient values by their standard errors and observing the probability mass corresponding to equal or more extreme values from both tails of a t-student distribution. Exact P values can be found in the **Supplementary Tables**.
The method for this sensitivity analysis is described in the **Supplementary Methods**.
AFR, African; AMR, Admixed American; EUR, European; MID, Middle Eastern; PGS, Polygenic Score.

Supplementary Figure 5 – Sensitivity analysis – "adjusting for T2D status in the statistical model": Effect of T2D and BMI polygenic scores on body weight changes

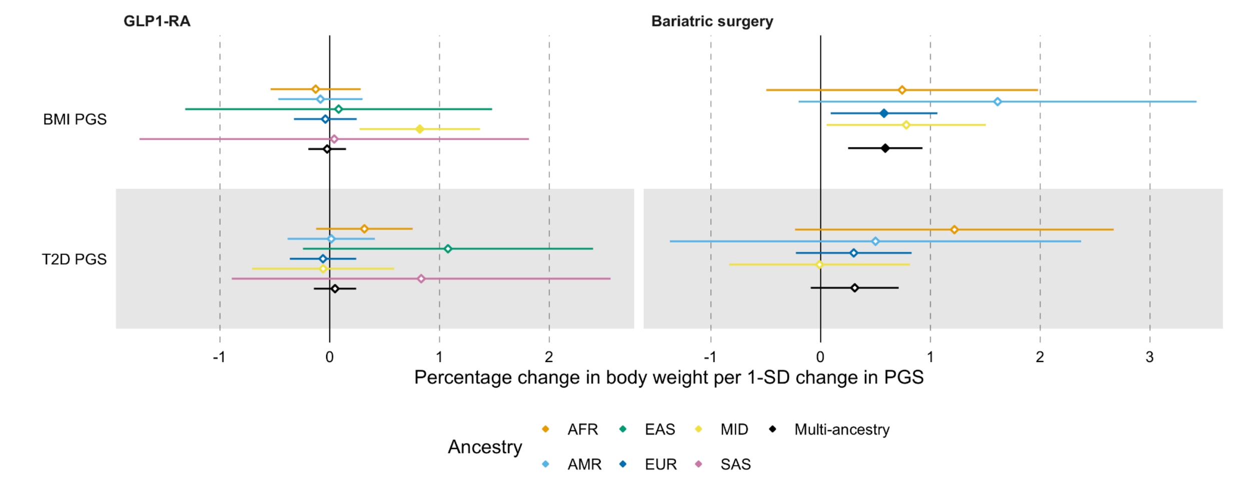

Supplementary Figure 5 | Sensitivity analysis – adjusting for T2D status in the statistical model: Effect of T2D and BMI polygenic scores on body weight changes associated with GLP1-RA treatment and bariatric surgery.
Ancestry-specific and multi-ancestry meta-analysis effect sizes for association between percentage change in body weight and PGS for BMI and type 2 diabetes. Dots represent the percentage change in body weight per one standard deviation change in PGS, error bars represent the 95% confidence interval. For both panels, coefficients are estimated from a linear regression model, separately for each genetic exposure. Full dots represent statistical significance at $P < 0.025$ (Bonferroni adjusted for 2 independent tests). P values are two-sided and were calculated by dividing the coefficient values by their standard errors and observing the probability mass corresponding to equal or more extreme values from both tails of a t-student distribution. Exact P values can be found in the **Supplementary Tables**.
The method for this sensitivity analysis is described in the **Supplementary Methods**.
AFR, African; AMR, Admixed American; EAS, East Asian; EUR, European; MID, Middle Eastern; SAS, South Asian; PGS, Polygenic Score.

Supplementary Figure 6 – Sensitivity analysis – “including only liraglutide/semaglutide in GLP1-RA analysis”: Average percentage change in body weight

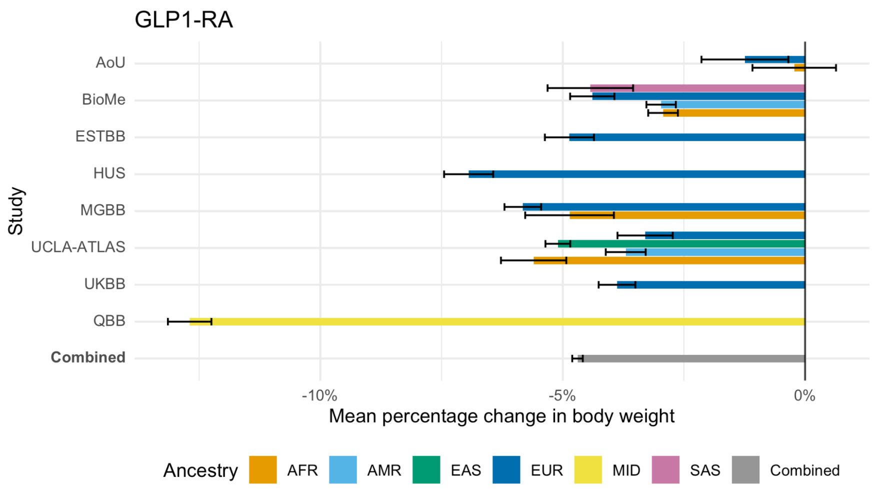

Supplementary Figure 6 | Sensitivity analysis – including only liraglutide/semaglutide in GLP1-RA analysis: Average percentage change in body weight for 4 431 GLP1-RA users.
Bars represent the mean percentage change in body weight within each study and ancestry, and the overall combined weighted mean change. Error bars represent standard errors.
Total N = 4 431, sample size per each cohort/ancestry can be found in the **Supplementary Methods**.
The method for this sensitivity analysis is described in the **Supplementary Methods**.
AoU, All of Us; ESTBB, Estonian Biobank; HUS, Helsinki Biobank; MGBB, Mass General Brigham Biobank; UCLA-ATLAS, Atlas Biobank; UKBB, UK Biobank; QBB, Qatar Biobank; AFR, African; AMR, Admixed American; EAS, East Asian; EUR, European; MID, Middle Eastern; SAS, South Asian

Supplementary Figure 7 – Sensitivity analysis – “including only liraglutide/semaglutide in GLP1-RA analysis”: Effect of T2D and BMI polygenic scores on body weight changes

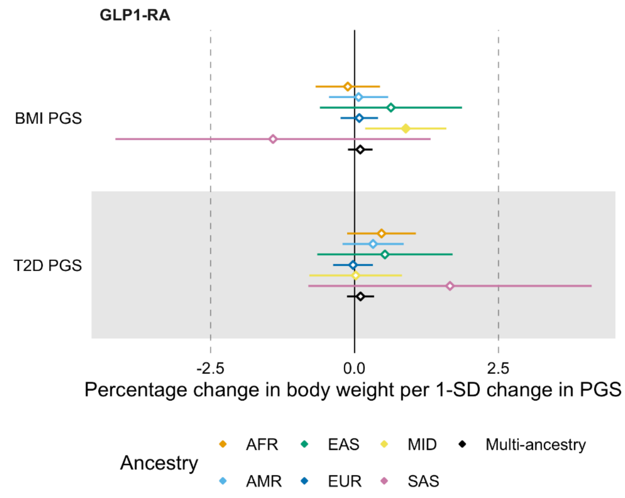

Supplementary Figure 7 | Sensitivity analysis – including only liraglutide/semaglutide in GLP1-RA analysis: Effect of T2D and BMI polygenic scores on body weight changes associated with GLP1-RA treatment and bariatric surgery.

Ancestry-specific and multi-ancestry meta-analysis effect sizes for association between percentage change in body weight and PGS for BMI and type 2 diabetes. Dots represent the percentage change in body weight per one standard deviation change in PGS, error bars represent the 95% confidence interval. Coefficients are estimated from a linear regression model, separately for each genetic exposure. Full dots represent statistical significance at $P < 0.025$ (Bonferroni adjusted for 2 independent tests). P values are two-sided and were calculated by dividing the coefficient values by their standard errors and observing the probability mass corresponding to equal or more extreme values from both tails of a t-student distribution. Exact P values can be found in the **Supplementary Tables**.

The method for this sensitivity analysis is described in the **Supplementary Methods**.

AFR, African; AMR, Admixed American; EAS, East Asian; EUR, European; MID, Middle Eastern; SAS, South Asian; PGS, Polygenic Score.

Supplementary Figure 8 – Sensitivity analysis – “stricter inclusion criteria for the BS analysis”: Average percentage change in body weight

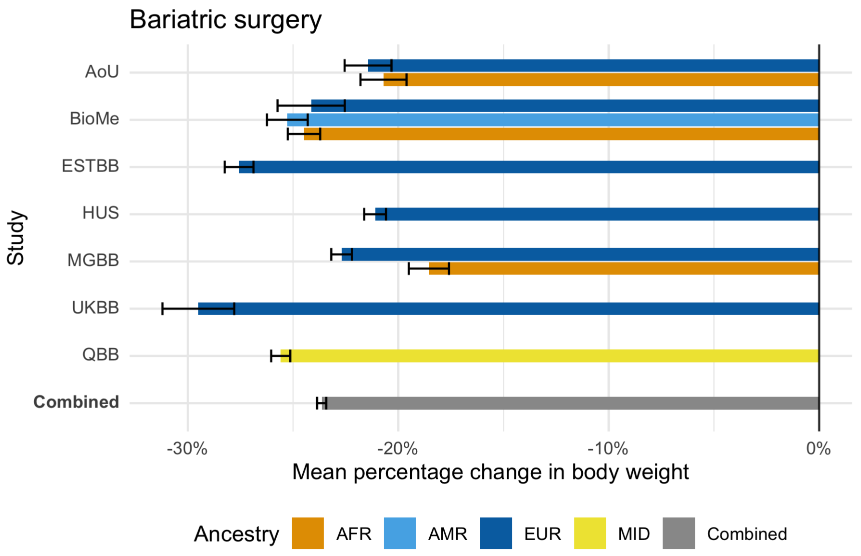

Supplementary Figure 8 | Sensitivity analysis – stricter inclusion criteria for the BS analysis: Average percentage change in body weight for 3 710 bariatric surgery patients.
Bars represent the mean percentage change in body weight within each study and ancestry, and the overall combined weighted mean change. Error bars represent standard errors.
Only participants with weight measurements available at least 30 days prior to surgery (from -365 days to -30 days relative to the day of surgery) were included.
Total N = 3 710, sample size per each cohort/ancestry can be found in the **Supplementary Methods**.
The method for this sensitivity analysis is described in the **Supplementary Methods**.
AoU, All of Us; BBSS, Bialystok Bariatric Surgery Study; ESTBB, Estonian Biobank; HUS, Helsinki Biobank; MGBB, Mass General Brigham Biobank; UKBB, UK Biobank; QBB, Qatar Biobank; AFR, African; AMR, Admixed American; EAS, East Asian; EUR, European; MID, Middle Eastern; SAS, South Asian

Supplementary Figure 9 – Sensitivity analysis – “stricter inclusion criteria for the BS analysis”: Effect of T2D and BMI polygenic scores on body weight changes

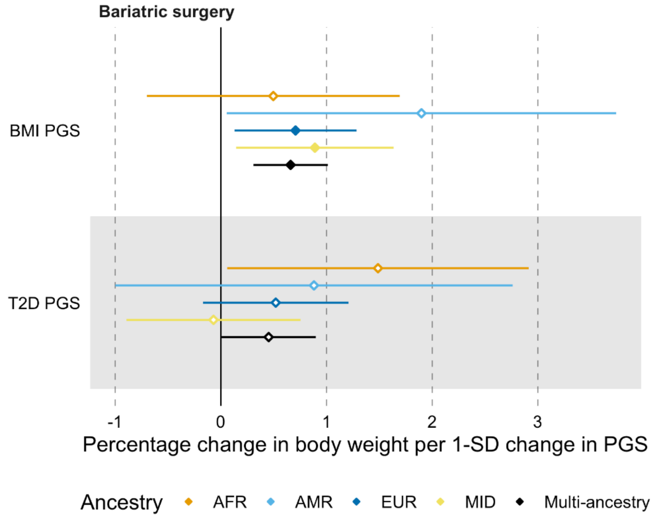

Supplementary Figure 9 | Sensitivity analysis – stricter inclusion criteria for the BS analysis: Effect of T2D and BMI polygenic scores on body weight changes associated with GLP1-RA treatment and bariatric surgery.
Ancestry-specific and multi-ancestry meta-analysis effect sizes for association between percentage change in body weight and PGS for BMI and type 2 diabetes. Dots represent the percentage change in body weight per one standard deviation change in PGS, error bars represent the 95% confidence interval. Coefficients are estimated from a linear regression model, separately for each genetic exposure. Full dots represent statistical significance at P < 0.025 (Bonferroni adjusted for 2 independent tests). P values are two-sided and were calculated by dividing the coefficient values by their standard errors and observing the probability mass corresponding to equal or more extreme values from both tails of a t-student distribution. Exact P values can be found in the **Supplementary Tables**.
Only participants with weight measurements available at least 30 days prior to surgery (from -365 days to -30 days relative to the day of surgery) were included.
The method for this sensitivity analysis is described in the **Supplementary Methods**.
AFR, African; AMR, Admixed American; EUR, European; MID, Middle Eastern; PGS, Polygenic Score.

Supplementary Figure 10 – Sensitivity analysis – “stratifying BS analysis by RYGB and SG procedures”: Average percentage change in body weight

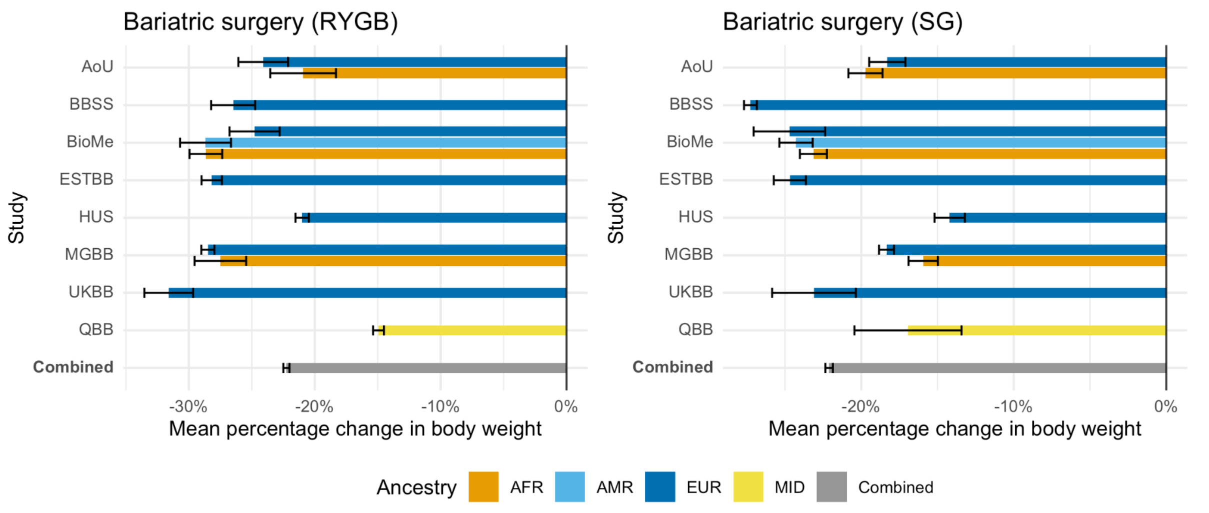

Supplementary Figure 10 | Sensitivity analysis – stratifying BS analysis by RYGB and SG procedures: Average percentage change in body weight for 4 036 bariatric surgery patients.
Bars represent the mean percentage change in body weight within each study and ancestry, and the overall combined weighted mean change. Error bars represent standard errors.
2 200 patients underwent BS with the RYGB procedure and 1 836 underwent BS with the SG procedure.
Total N = 4 036, sample size per each cohort/ancestry can be found in the Supplementary Methods.
The method for this sensitivity analysis is described in the **Supplementary Methods**.
RYGB, Roux-en-Y gastric bypass; SG, Sleeve gastrectomy; AoU, All of Us; BBSS, Bialystok Bariatric Surgery Study; ESTBB, Estonian Biobank; HUS, Helsinki Biobank; MGBB, Mass General Brigham Biobank; UKBB, UK Biobank; QBB, Qatar Biobank; AFR, African; AMR, Admixed American; EUR, European; MID, Middle Eastern

Supplementary Figure 11 – Sensitivity analysis – “stratifying BS analysis by RYGB and SG procedures”: Effect of T2D and BMI polygenic scores on body weight changes

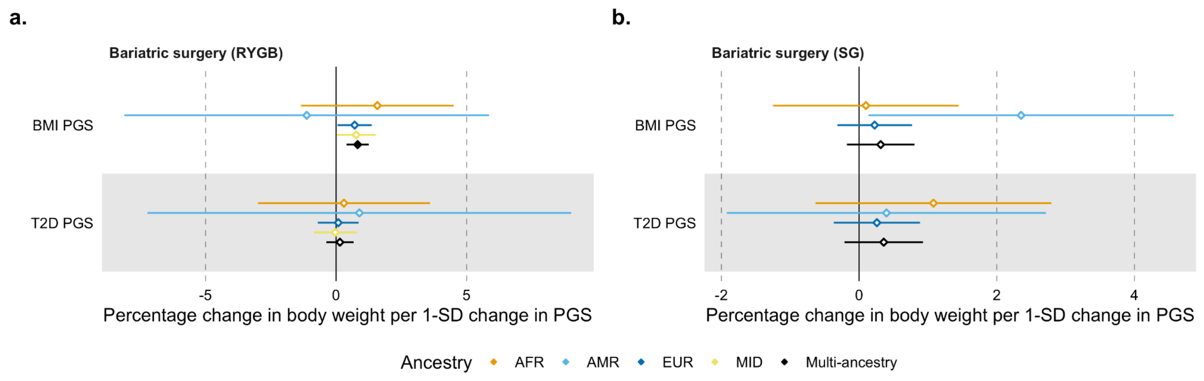

Supplementary Figure 11 | Sensitivity analysis – stratifying BS analysis by RYGB and SG procedures): Effect of T2D and BMI polygenic scores on body weight changes associated with bariatric surgery.
Ancestry-specific and multi-ancestry meta-analysis effect sizes for association between percentage change in body weight and PGS for BMI and type 2 diabetes. Dots represent the percentage change in body weight per one standard deviation change in PGS, error bars represent the 95% confidence interval.
a. Patients undergoing Roux-en-Y gastric bypass (RYGB)
b. Patients undergoing Sleeve gastrectomy (SG)
For both panels, coefficients are estimated from a linear regression model, separately for each genetic exposure. Full dots represent statistical significance at $P < 0.025$ (Bonferroni adjusted for 2 independent tests). P values are two-sided and were calculated by dividing the coefficient values by their standard errors and observing the probability mass corresponding to equal or more extreme values from both tails of a t-student distribution. Exact P values can be found in the **Supplementary Tables**.
The method for this sensitivity analysis is described in the **Supplementary Methods**.
AFR, African; AMR, Admixed American; EUR, European; MID, Middle Eastern; PGS, Polygenic Score.

Supplementary Figure 12 – Sensitivity analysis – "model without adjustment for baseline weight (W0)": Effect of T2D and BMI polygenic scores on body weight changes

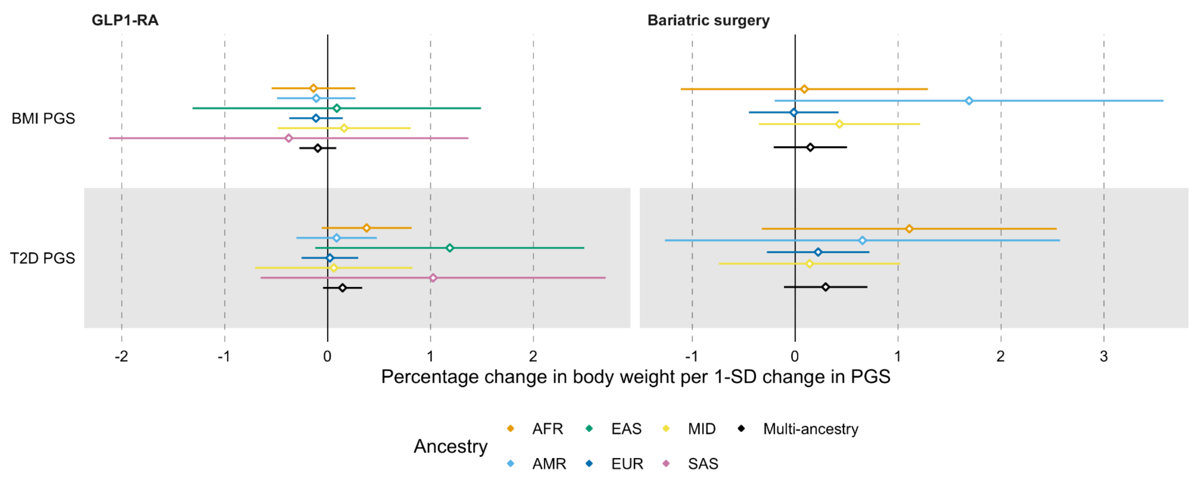

Supplementary Figure 12 | Sensitivity analysis – model without baseline weight (W0): Effect of T2D and BMI polygenic scores on body weight changes associated with GLP1-RA treatment and bariatric surgery.
Ancestry-specific and multi-ancestry meta-analysis effect sizes for association between percentage change in body weight and PGS for BMI and type 2 diabetes. Dots represent the percentage change in body weight per one standard deviation change in PGS, error bars represent the 95% confidence interval. For both panels, coefficients are estimated from a linear regression model, separately for each genetic exposure. Full dots represent statistical significance at $P < 0.025$ (Bonferroni adjusted for 2 independent tests). P values are two-sided and were calculated by dividing the coefficient values by their standard errors and observing the probability mass corresponding to equal or more extreme values from both tails of a t-student distribution. Exact P values can be found in the **Supplementary Tables**.
The method for this sensitivity analysis is described in the **Supplementary Methods**.
AFR, African; AMR, Admixed American; EAS, East Asian; EUR, European; MID, Middle Eastern; SAS, South Asian; PGS, Polygenic Score.
