# Supplementary material for: Association between plausible genetic factors and weight loss from GLP1-RA and bariatric surgery
Source: Nat Med. 2025 Apr 18;31(7):2269–76. doi: 10.1038/s41591-025-03645-3 (PMC12283387; doi:10.1038/s41591-025-03645-3)
Supplement: Supplementary file 2 — Reporting Summary [file 41591_2025_3645_MOESM2_ESM.pdf]

nature portfolio

Corresponding author(s): Andrea Ganna, PhD

Last updated by author(s): Mar 3, 2025

Reporting Summary

Nature Portfolio wishes to improve the reproducibility of the work that we publish. This form provides structure for consistency and transparency in reporting. For further information on Nature Portfolio policies, see our Editorial Policies and the Editorial Policy Checklist.

Statistics

For all statistical analyses, confirm that the following items are present in the figure legend, table legend, main text, or Methods section.

n/a	Confirmed	
☐	☒	The exact sample size (*n*) for each experimental group/condition, given as a discrete number and unit of measurement
☐	☒	A statement on whether measurements were taken from distinct samples or whether the same sample was measured repeatedly
☐	☒	The statistical test(s) used AND whether they are one- or two-sided<br>*Only common tests should be described solely by name; describe more complex techniques in the Methods section.*
☐	☒	A description of all covariates tested
☐	☒	A description of any assumptions or corrections, such as tests of normality and adjustment for multiple comparisons
☐	☒	A full description of the statistical parameters including central tendency (e.g. means) or other basic estimates (e.g. regression coefficient) AND variation (e.g. standard deviation) or associated estimates of uncertainty (e.g. confidence intervals)
☐	☒	For null hypothesis testing, the test statistic (e.g. *F*, *t*, *r*) with confidence intervals, effect sizes, degrees of freedom and *P* value noted<br>*Give P values as exact values whenever suitable.*
☒	☐	For Bayesian analysis, information on the choice of priors and Markov chain Monte Carlo settings
☒	☐	For hierarchical and complex designs, identification of the appropriate level for tests and full reporting of outcomes
☐	☒	Estimates of effect sizes (e.g. Cohen's *d*, Pearson's *r*), indicating how they were calculated

Our web collection on statistics for biologists contains articles on many of the points above.

Software and code

Policy information about availability of computer code

Data collection	No software was used for data collection.

Data analysis

The study utilized previously published analysis tools as described in the Methods section of the manuscript. Additional code used for these analyses is available at https://github.com/dsgelab/glp1-bs-genetics/

Tools/Software:
Plink v2
R (version 4.0 or higher)
R packages:
tidyverse (v 2.0.0)
ggplot2 (v 3.5.5)
data.table (v 1.15.4)
meta (v 7.0.0)
ggforestplot (v 0.1.0)
readxl (v 1.4.3)
cowplot (v 1.1.3)
ggtext (v 0.1.2)
patchwork (v 1.2.0)

For manuscripts utilizing custom algorithms or software that are central to the research but not yet described in published literature, software must be made available to editors and reviewers. We strongly encourage code deposition in a community repository (e.g. GitHub). See the Nature Portfolio guidelines for submitting code & software for further information.

Data

Policy information about availability of data

All manuscripts must include a data availability statement. This statement should provide the following information, where applicable:

- Accession codes, unique identifiers, or web links for publicly available datasets
- A description of any restrictions on data availability
- For clinical datasets or third party data, please ensure that the statement adheres to our policy

The Helsinki biobank can provide access for research projects within the scope regulated by the Finnish Biobank Act, which is research utilizing the biobank samples or data for the purposes of promoting health, understanding the mechanisms of disease or developing products and treatment practices used in health and medical care.
De-identified data of the MGBB that supports this study is available from the MGB Biobankportal at https://www.massgeneralbrigham.org/en/research-and-innovation/participate-in-research/biobank/for-researchers. Restrictions apply to the availability of these data, which are available to MGB-affiliated researchers via a formal application.
UK Biobank data are available through a procedure described at http://www.ukbiobank.ac.uk/using-the-resource/.
Registered researchers whose institutions have Data Use and Registration Agreements in place with All of Us that include the Controlled Tier can access genomic data.
Clinical and genotype data from UCLA ATLAS Community Health Initiative patients, de-identified for research purposes, are accessible to UCLA-approved researchers through the Discovery Data Repository (DDR).
BioMe data is available through a process described at https://icahn.mssm.edu/research/ipm/programs/biome-biobank/researcher-faqs.
Pseudonymised data and/or biological samples can be accessed for research and development purposes in accordance with the Estonian Human Genome Research Act. To access data, the research proposal must be approved by the Scientific Advisory Committee of the Estonian Biobank as well as by the Estonian Committee on Bioethics and Human Research. For more details on data access and relevant documents, please see https://genomics.ut.ee/en/content/estonian-biobank#dataaccess.
For access to the Bialystok Bariatric Surgery Study please refer to the Medical University of Bialystok.
The data and biosamples collected or generated by QBB will be made available to researchers employed within or otherwise contractually bound to public and private institutions that conduct scientific research and that meet the requirements detailed in the Qatar Biobank Research Access policy.
The PGS weights used in this study are available in Weissbrod et al. 2022 and Thompson et al. 2022.

Research involving human participants, their data, or biological material

Policy information about studies with human participants or human data. See also policy information about sex, gender (identity/presentation), and sexual orientation and race, ethnicity and racism.

Reporting on sex and gender

Sex was used as a covariate in all of the analysis and no analyses were run separately in each sex. Sex was geneticallly determined in all cohorts. More information for each cohort is available in the Methods section.

Reporting on race, ethnicity, or other socially relevant groupings

Each cohort was analyzed separately, stratified by major continental ancestry groups, and the effects were subsequently meta-analyzed. For multi-ancestry biobanks (AoU, BioMe, MGBB, UCLA-ATLAS), we also assessed whether the effects of GLP1-RA and bariatric surgery (BS)-associated weight loss differed significantly across genetically defined continental ancestry groups.
A detailed description of ancestry computation methods for each cohort is available in the Methods section. Briefly, ancestry determination across cohorts utilized a combination of genotype-based analyses and self-reported race/ethnicity, where available. Methods included principal component analysis, clustering algorithms, and comparisons to reference populations such as those from the 1000 Genomes Project or gnomAD. Some cohorts also employed admixture models or network-based clustering to refine ancestry assignments, aligning participants with reference ancestry groups or superpopulations.

Population characteristics

Our study consists of 9 cohorts (Helsinki University Hospital (HUS, Finland; N = 633), Estonian Biobank (ESTBB, Estonia; N =

Population characteristics	464), UK Biobank (UKBB, United Kingdom; N = 810), All of Us (AoU, USA; N = 559), BioMe (BioMe, USA; N = 2,170), Mass General Brigham Biobank (MGBB, USA; N = 2,141), Atlas Biobank (UCLA-ATLAS, USA; N = 1,445), Qatar Biobank (QBB, Qatar; N = 2,383), Bialystok Bariatric Surgery Study (BBSS, Poland, N = 355).) Population Characteristics, such as Age, Sex, type 2 diabetes prevalence and the proportion of semaglutide use can be found in Table 1 of the main manuscript text. Please find further relevant information in the cohort descriptions in the Methods section.
Recruitment	Please find the relevant information in the cohort descriptions in the Methods section.
Ethics oversight	*Identify the organization(s) that approved the study protocol.*

Note that full information on the approval of the study protocol must also be provided in the manuscript.

Field-specific reporting

Please select the one below that is the best fit for your research. If you are not sure, read the appropriate sections before making your selection.

☒ Life sciences ☐ Behavioural & social sciences ☐ Ecological, evolutionary & environmental sciences

For a reference copy of the document with all sections, see nature.com/documents/nr-reporting-summary-flat.pdf

Life sciences study design

All studies must disclose on these points even when the disclosure is negative.

Sample size	A total sample size of 10 960 individuals (GLP1-RA users N = 6750 and bariatric surgery patients N = 4 210) across all cohorts was included in the study. Sample size per each cohort/ancestry can be found in Table 1. Sample sizes for each analysis (primary analysis and sensitivity analyses) were determined by the current biobank sizes of the participating cohorts based on the study protocol (main manuscript and supplementary information).
Data exclusions	Inclusion and exclusion criteria are listed in the Methods section of the manuscript.
Replication	The study was successfully conducted across 9 distinct cohorts. The findings are described in the manuscript.
Randomization	This point is not applicable here as the analyses were performed in population based biobanks (observational data).
Blinding	This point is not applicable here as the data points were collected from biobank participants medical records and blood samples (genotype data), and all the data were collected before any of the analyses of this study were planned. The data was pseudonymized, and the participants could not be identified nor be interacted with.

Reporting for specific materials, systems and methods

We require information from authors about some types of materials, experimental systems and methods used in many studies. Here, indicate whether each material, system or method listed is relevant to your study. If you are not sure if a list item applies to your research, read the appropriate section before selecting a response.

Materials & experimental systems

n/a	Involved in the study	
☒	☐	Antibodies
☒	☐	Eukaryotic cell lines
☒	☐	Palaeontology and archaeology
☒	☐	Animals and other organisms
☐	☒	Clinical data
☒	☐	Dual use research of concern
☒	☐	Plants

Methods

n/a	Involved in the study	
☒	☐	ChIP-seq
☒	☐	Flow cytometry
☒	☐	MRI-based neuroimaging

Clinical data

Policy information about clinical studies

All manuscripts should comply with the ICMJE guidelines for publication of clinical research and a completed CONSORT checklist must be included with all submissions.

Clinical trial registration	N/A
Study protocol	Details on the study protocol can be found in the methods section of the manuscript.

Data collection	Detailed cohort descriptions can be found in the supplementary material.
Outcomes	Absolute and relative weight change were defined as primary outcomes. For further information please refer to the methods and supplementary methods.

Plants

Seed stocks	*Report on the source of all seed stocks or other plant material used. If applicable, state the seed stock centre and catalogue number. If plant specimens were collected from the field, describe the collection location, date and sampling procedures.*
Novel plant genotypes	*Describe the methods by which all novel plant genotypes were produced. This includes those generated by transgenic approaches, gene editing, chemical/radiation-based mutagenesis and hybridization. For transgenic lines, describe the transformation method, the number of independent lines analyzed and the generation upon which experiments were performed. For gene-edited lines, describe the editor used, the endogenous sequence targeted for editing, the targeting guide RNA sequence (if applicable) and how the editor was applied.*
Authentication	*Describe any authentication procedures for each seed stock used or novel genotype generated. Describe any experiments used to assess the effect of a mutation and, where applicable, how potential secondary effects (e.g. second site T-DNA insertions, mosiacism, off-target gene editing) were examined.*
